# Contribution of Gli1^+^ Adventitial Stem Cells to Smooth Muscle Cells in Atherosclerosis and Vascular Injury

**DOI:** 10.1002/advs.202512897

**Published:** 2025-12-03

**Authors:** Haixiao Wang, Xiuzhen Huang, Jialing Mou, Enci Wang, Yan Li, Wenjuan Pu, Yao Xie, Xiaoling Guo, Lixin Wang, Maoping Chu, Bin Zhou, Chao Niu

**Affiliations:** ^1^ Pediatric Research Institute Pediatric Discipline Group The Second Affiliated Hospital and Yuying Children's Hospital of Wenzhou Medical University Wenzhou 325000 China; ^2^ Children's Heart Center The Second Affiliated Hospital and Yuying Children's Hospital of Wenzhou Medical University Wenzhou 325000 China; ^3^ Zhejiang Provincial Clinical Research Center for Pediatric Disease Wenzhou 325000 China; ^4^ New Cornerstone Science Laboratory Key Laboratory of Multi‐Cell Systems Shanghai Institute of Biochemistry and Cell Biology Center for Excellence in Molecular Cell Science Chinese Academy of Sciences University of Chinese Academy of Sciences Shanghai 200031 China; ^5^ Department of Vascular Surgery Zhongshan Hospital Fudan University Shanghai 200031 China; ^6^ Shandong Laboratory of Yantai Drug Discovery Bohai Rim Advanced Research Institute for Drug Discovery Yantai Shandong 264199 China; ^7^ State Key Laboratory of Drug Research Shanghai Institute of Materia Medica Chinese Academy of Sciences Shanghai 201203 China; ^8^ Department of Cardiology Second Affiliated Hospital Zhejiang University School of Medicine Hangzhou Zhejiang 310009 China; ^9^ Scientific Research Department The Second Affiliated Hospital and Yuying Children's Hospital of Wenzhou Medical University Wenzhou 325000 China

**Keywords:** atherosclerosis, fate‐mapping, lineage tracing, stem cell, vascular smooth muscle cell

## Abstract

Adventitial stem cells (ASCs), identified by Gli1 expression, have been proposed as key contributors to the vascular smooth muscle cell (SMC) population during atherosclerosis development. However, their precise role remains a subject of debate. To clarify this, a Gli1‐CreER lineage tracing tool to track these cells in an atherosclerosis model is initially used. This fate‐mapping studies revealed that Gli1^+^ cells contribute to a small subset of SMCs. To definitively differentiate between the true lineage transformation and potential ectopic labeling by Gli1‐CreER in SMCs, a dual recombinase‐mediated genetic strategy to label Gli1^+^ ASCs while eliminating ectopic labeling in SMCs is developed. The results from this dual lineage tracing approach demonstrated that Gli1^+^ ASCs do not contribute to the SMC population in atherosclerotic plaques. Instead, Gli1^+^ ASCs differentiate into a significant portion of SMCs after vascular anastomosis injury, suggesting their role is context‐dependent. These findings challenge current paradigms and highlight the need to reconsider cellular targets for therapeutic interventions in atherosclerosis.

## Graphical abstract

Gli1^+^ adventitial stem cells (ASCs) have been thought to generate smooth muscle cells (SMCs) in atherosclerosis. Using a dual‐recombinase lineage tracing to exclude ectopic labeling, Wang et al. found that Gli1^+^ ASCs do not contribute to SMCs in atherosclerotic plaques. Instead, Gli1^+^ ASCs contribute to a substantial portion of SMCs after vascular anastomosis injury, indicating a context‐dependent role. These results challenge existing views and urge a re‐evaluation of therapeutic targets for atherosclerosis.

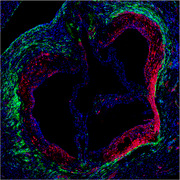

## 1 Introduction

Atherosclerosis is globally recognized as the leading inflammatory cardiovascular disease.^[^ ^1^, ^2^ ^]^ It is principally marked by the progressive deposition of lipids and foam cells in the subintimal space of the aorta, which culminates in the formation of atheromatous plaques.^[^ ^3^ ^]^ Such accumulations significantly diminish the diameter of the vascular lumen, consequently elevating blood pressure and inducing widespread death of vascular smooth muscle cells (SMCs). These SMCs, which reside in the arterial medial layer, are identifiable by specific molecular markers including smooth muscle actin (SMA), SM22α, smooth muscle myosin heavy chain (SM‐MHC, Myh11), and calponin (CNN1).^[^ ^4^, ^5^, ^6^, ^7^, ^8^ ^]^ These markers not only help in identifying SMCs but also indicate their functional state and health in the vascular system. As SMC death occurs, the structural and mechanical stability of the vessel wall is compromised, exacerbating the risk and severity of atherosclerotic events. SMCs play a pivotal role in regulating vascular tone, partially governing blood pressure and overall vessel diameter through their contractile capabilities.^[^ ^4^, ^9^ ^]^ In atherosclerosis, SMCs help generate a fibrous cap, which enhances plaque stability.^[^ ^10^, ^11^ ^]^ However, as atherosclerosis progresses, the apoptosis of these SMCs weakens the fibrous cap, leading to plaque instability, rupture, and potential thrombosis.^[^ ^12^, ^13^, ^14^, ^15^ ^]^ Such events can lead to severe outcomes, including myocardial infarction and strokes. Therefore, understanding the origins and development of vascular SMCs within atherosclerotic plaques is essential for advancing our knowledge of the pathogenesis of atherosclerosis.

The vascular adventitia primarily comprises fibroblasts, along with various other cell types.^[^ ^16^, ^17^, ^18^ ^]^ It has been reported that vascular adventitial fibroblasts can infiltrate atherosclerotic plaque and differentiate into SMA‐positive myofibroblasts.^[^ ^19^ ^]^ Additionally, adventitial fibroblasts may serve as a source of SMCs in other vascular injury models.^[^ ^20^ ^]^ Previous research has underscored the importance of adventitial stem cells (ASCs) in generating SMCs. In a seminal study by Hu et al., a population of Sca1^+^ cells were identified within the aortic root of the ApoE^–/–^ atherosclerotic mouse model, indicating that these cells could differentiate into SMCs upon transplantation into grafts.^[^ ^21^ ^]^ Subsequently, Tsai et al. employed in vivo transplantation techniques to further substantiate the role of Sca1^+^ cells in contributing to SMC formation.^[^ ^22^ ^]^ Additionally, genetic lineage tracing of Sca1^+^ cells revealed their contribution to SMC formation in the context of acute vascular anastomosis injury,^[^ ^7^ ^]^ but not in the LDLR^–/–^ mouse model.^[^ ^23^ ^]^ Recent studies have demonstrated a marked reduction in the Sca1^+^ cell population at the aortic root of *Shh* ^–/–^ mice, suggesting that the Shh signaling pathway regulates these cells within the vascular adventitia.^[^ ^24^ ^]^

The Shh pathway is crucial in vertebrate embryonic development, affecting cell proliferation and differentiation,^[^ ^25^, ^26^, ^27^ ^]^ and its disruption can lead to developmental abnormalities and various diseases.^[^ ^28^, ^29^ ^]^ Gli1, a downstream transcription factor of the Shh pathway,^[^ ^30^ ^]^ serves as a marker for mesenchymal stem cells.^[^ ^31^, ^32^, ^33^, ^34^ ^]^ Gli1^+^ cells in the perivascular space are crucial for maintaining tissue homeostasis and aiding in injury repair.^[^ ^31^ ^]^ Kramann and colleagues reported the involvement of perivascular Gli1^+^ cells in fibrosis progression across multiple organs, including the kidney, liver, lung, and heart, through the generation of myofibroblasts.^[^ ^35^ ^]^ With a high presence of Shh signaling in the vascular adventitia, Gli1^+^ ASCs were reported to differentiate into SMCs during vascular injury.^[^ ^36^, ^37^ ^]^ Kramann et al. further reported that Gli1^+^ ASCs are progenitors of SMCs, contributing to neointima formation and arterial repair.^[^ ^36^ ^]^ These findings highlight the potential of Gli1^+^ ASCs to differentiate into SMCs in response to arterial injury. However, unanswered questions persist in the field, including the distribution of Gli1^+^ ASCs across different aortic regions under normal conditions, the potential caveat of ectopic lineage tracing of a subset of SMCs by Gli1‐CreER during lineage tracing, and the involvement of Gli1^+^ ASCs in the generation of de novo SMCs under atherosclerotic conditions.

In this study, we explored the cell fate plasticity of Gli1^+^ ASCs in atherosclerosis and vascular injury. Initially, we used a Gli1‐CreER to trace Gli1^+^ ASCs, but this inadvertently labeled a small subset of SMCs under normal conditions. To resolve this, we next employed a dual recombinase‐mediated intersectional genetic strategy to specifically mark Gli1^+^ ASCs. Fate mapping results reveal that Gli1^+^ ASCs do not contribute to SMC formation during homeostasis or within atherosclerotic plaques. Instead, these cells generate de novo SMCs following arterial anastomosis injury, underscoring their critical role in arterial repair and regeneration. This research introduces an intersectional genetic system for precise analysis of ASC cell fates and functions across various conditions including arterial homeostasis, acute injury, and atherosclerosis. Our genetic lineage tracing of Gli1^+^ ASCs with enhanced precision provides robust evidence of their adaptability in artery repair, regeneration, and diseases.

## 2 Results

### 2.1 Characterization of Gli1⁺ ASCs in the Aorta Using *Gli1‐CreER* Mice

**Figure 1:**
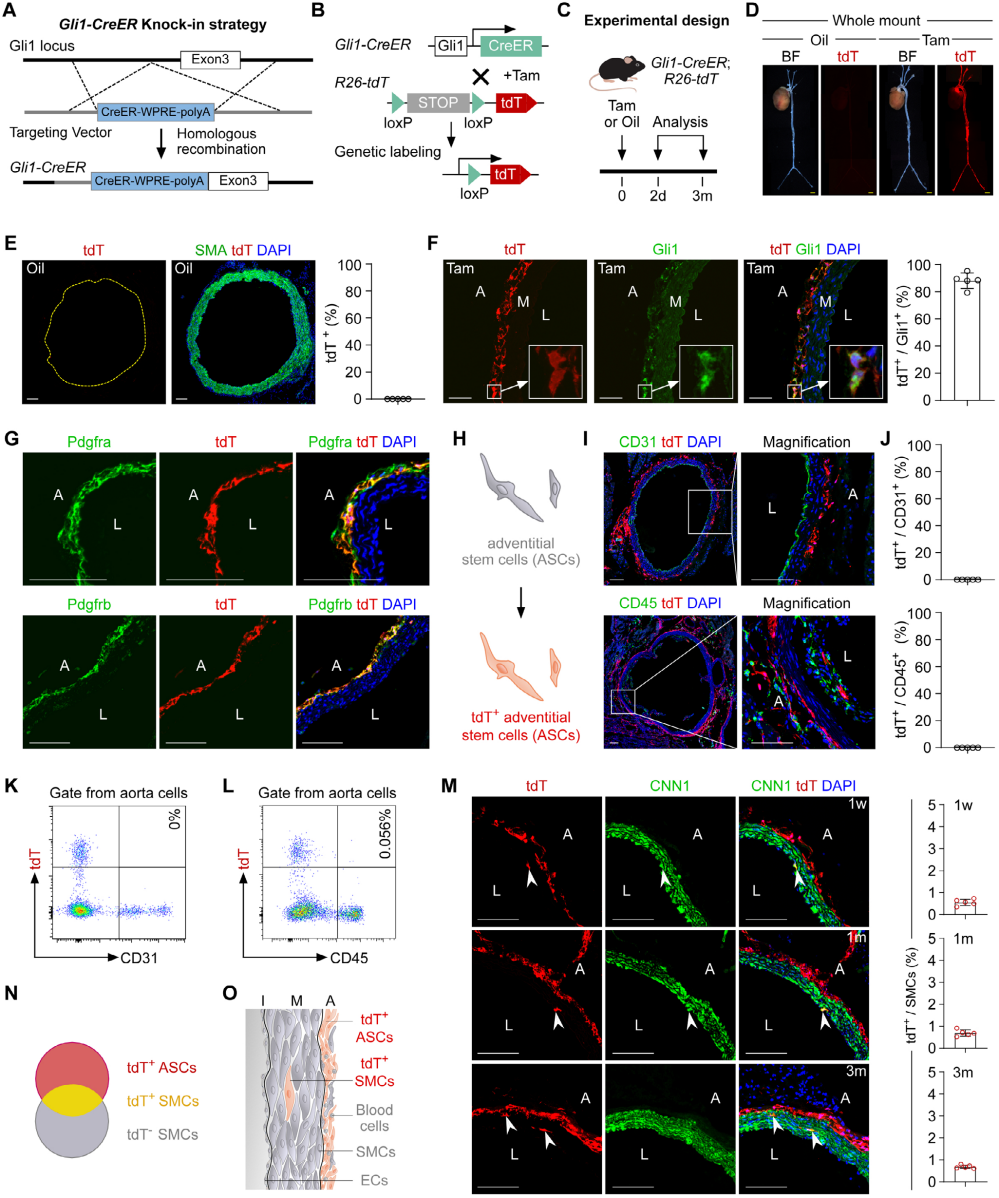
Characterization of Gli1^+^ cells during homeostasis. A) Schematic diagram of the *Gli1‐CreER* mice knock‐in strategy. B) A schematic showing the lineage tracing strategy. C) A schematic showing the experimental design. Tam: tamoxifen. Oil: no Tam control. D) Whole‐mount bright‐field (BF) and epifluorescence microscopy images of aortas from *Gli1‐CreER;R26‐tdT* mice. E,F) Immunostaining for tdT and SMA (E), or Gli1 (F) on aortic sections (E: aortic arch, F: femoral artery). A: adventitia, M: media, L: lumen, *n* = 5. G–J) Immunostaining for Pdgfra, Pdgfrb (G), CD31, and CD45 (I) in conjunction with tdT on aortic sections (CD31: descending aorta, CD45: aortic root), *n* = 5. A cartoon for (H) indicates that the ASCs surrounding the aorta were labeled by Gli1‐CreER. The statistical analysis in (J) show the quantification of the percentage of endothelial (CD31^+^) or immune (CD45^+^) cells that express tdT. K,L) Flow cytometry analysis showing expression of CD31 or CD45 in tdT^+^ cells. M) Immunostaining for CNN1 and tdT on aortic sections (descending aorta) at different time points after Tam treatment, *n* = 5. N,O) The Venn diagram and cartoon summarizing the labeling of Gli1^+^ cells in the aorta. ASCs: adventitial stem cells, SMCs: smooth muscle cells, ECs: endothelial cells. I: intima, M: media, A: adventitia. White scale bars: 100 µm, yellow scale bars: 1000 µm.

To characterize Gli1⁺ ASCs in the aorta, we generated *Gli1‐CreER* mice by inserting the CreER‐WPRE‐polyA cassette into the translational start codon of the Gli1 gene using CRISPR/Cas9 (**Figure** **1**A). For in vivo labeling of Gli1⁺ ASCs, *Gli1‐CreER* mice were crossed with *Rosa26‐loxP‐stop‐loxP‐tdTomato* (*R26‐tdT*) reporter mice.^[^ ^38^ ^]^ In *Gli1‐CreER;R26‐tdT* mice, tamoxifen treatment induces the nuclear translocation of CreER, resulting in Cre‐loxP recombination and permanent tdT expression in Gli1‐expressing cells and their progeny, thereby enabling indelible lineage tracing of Gli1⁺ ASCs (Figure 1B). For this characterization, *Gli1‐CreER;R26‐tdT* mice (aged 8–10 weeks) were treated with tamoxifen or oil, and tissues were harvested at 2 days or 3 months after treatment (Figure 1C). Whole‐mount fluorescence imaging of aortas and other organs collected 2 days after tamoxifen treatment revealed robust tdT fluorescence, whereas no signal was detected in tissues fromoil‐treated mice (Figure 1D; Figure S1A–D, Supporting Information). Immunostaining for tdT and SMA on aortic sections from oil‐treated mice confirmed the absence of reporter leakiness without tamoxifen induction (Figure 1E; Figure S2H–J, Supporting Information). No such leakiness was observed in other organs (Figure S1D, Supporting Information). Immunostaining for tdT and Gli1 on aortic sections from tamoxifen‐treated mice showed that ≈90% of Gli1⁺ cells in the adventitia were tdT‐positive, confirming the high labeling efficiency of the Gli1‐CreER system (Figure 1F). To assess Hedgehog signaling activity, we collected descending aortic samples from 5‐week‐old *Gli1‐CreER* heterozygous mice (experimental group) and their wild‐type littermates (controls). RNA isolated from the vascular adventitia of these samples was subjected to qPCR analysis for key pathway genes (Sufu, Smo, Ptch1, and Gli1). Although Sufu and Smo expression levels were comparable between the groups, the experimental group exhibited reduced mRNA levels of Gli1 and Ptch1 (Figure S1E, Supporting Information), indicating a partial attenuation of Hedgehog signaling in the aortic adventitia of *Gli1‐CreER* heterozygous mice. Nevertheless, immunostaining revealed no significant differences in Pdgfra expression in the vascular adventitia during aortic development between *Gli1‐CreER* and wild‐type mice (Figure S1F, Supporting Information).

To identify the cell types labeled by Gli1‐CreER, we performed immunostaining for mesenchymal and fibroblast markers. We found that tdT⁺ cells in the adventitia were positive for Pdgfra and Pdgfrb (Figure 1G,H), consistent with a previous study.^[^ ^36^ ^]^ Co‐immunostaining revealed no tdT expression in endothelial cells or blood cells, marked by CD31 and CD45, respectively (Figure 1I,J), a finding corroborated by flow cytometric analysis (Figure 1K,L). A previous study reported that Gli1⁺ ASCs reside in the adventitia during homeostasis and migrate to the media to differentiate into SMCs under pathological conditions.^[^ ^36^ ^]^ This finding prompted us to investigate whether Gli1⁺ ASCs in the media resulted from migration or ectopic labeling by Gli1‐CreER. We collected aortas at various time points after tamoxifen treatment for immunostaining and found that tdT‐positive cells expressing SMC markers appeared in the vascular media at 1 week (0.55%), 1 month (0.68%), and 3 months (0.69%) (Figure 1M). This result indicates that Gli1‐CreER also labels a small subset of SMCs in the aorta. Thus, although Gli1‐CreER primarily targets Gli1⁺ ASCs, it can also ectopically label SMCs in the media (Figure 1N,O).

### 2.2 Gli1⁺ ASCs Contribute to SMCs during Anastomosis Injury

We next investigated the regional specificity of Gli1‐CreER labeling across different segments of the aorta. Aortas were collected from *Gli1‐CreER;R26‐tdT* mice two weeks after tamoxifen treatment (Figure S2A, Supporting Information) and segmented into four regions for detailed analysis: the aortic root (AR), aortic arch (AA), descending aorta (DA), and femoral artery (FA) (Figure S2B, Supporting Information). Immunostaining for SMA and tdT across these sections revealed the presence of SMA⁺tdT⁺ cells in the AR, AA, and DA, but not in the FA (Figure S2C, Supporting Information). Quantitative analysis showed that ≈0.5% of SMA⁺ cells in the media of the AR, AA, and DA were tdT‐positive, with no detectable tdT⁺ cells in the FA (Figure S2D, Supporting Information). This finding was further validated by flow cytometric analysis, which confirmed the genetic labeling of a subset of SM22⁺ cells in the AR, AA, and DA, but not in the FA (Figure S2E,F, Supporting Information). Collectively, these results indicate that whereas Gli1‐CreER specifically targets ASCs in the FA, it also ectopically labels a small proportion of SMCs in other aortic segments (Figure S2G, Supporting Information).

**Figure 2:**
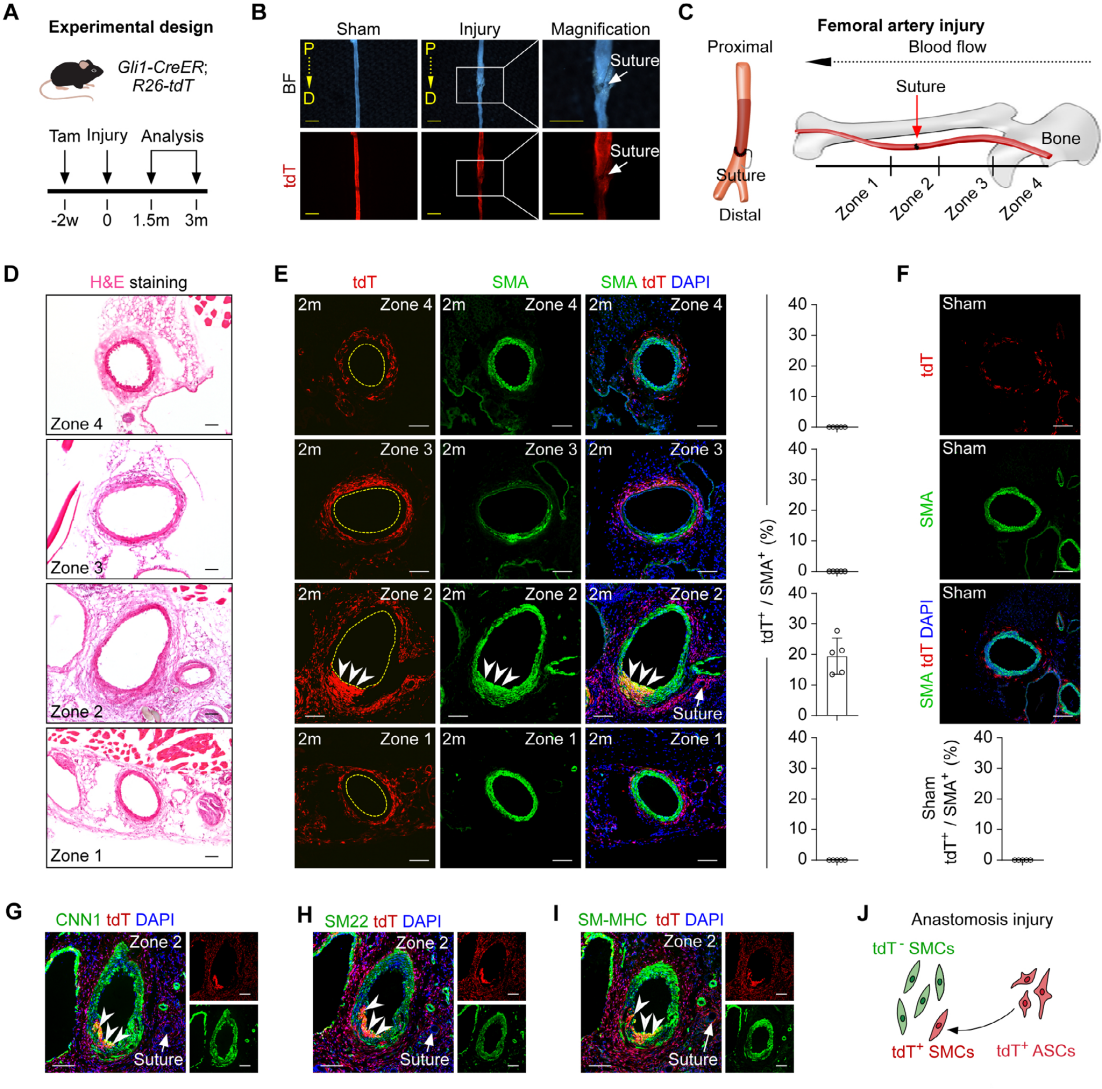
Gli1^+^ ASCs differentiate into SMCs after anastomosis injury. A) Schematic diagram of the experimental design. B) Whole‐mount bright‐field and epifluorescence images of sham FA and injury FA. P: proximal, D: distal. The arrow indicates the anastomosis suture site. C) A cartoon showing the division of FA according to the position of the vascular anastomosis suture. D) H&E staining on different zones of the femoral artery. E–I) Immunostaining for tdT with the SMC antibodies on injury (E, G–I) and sham FA sections (F), SMA (E and F), CNN1 (G), SM22 (H), and SM‐MHC (I). Quantification of the percentage of SMCs expressing tdT is shown in (E and F), *n* = 5. J) A cartoon showing that tdT^+^ ASCs differentiate into SMCs under vascular anastomosis injury. White and black scale bars: 100 µm, yellow scale bars: 1000 µm.

Given the absence of ectopic SMC labeling by Gli1‐CreER in the femoral artery (Figure S2G, Supporting Information), we investigated whether Gli1⁺ ASCs could differentiate into SMCs in the FA following arterial anastomosis surgery—a procedure that induces substantial death of resident SMCs at the injury site.^[^ ^7^, ^39^ ^]^ Adult *Gli1‐CreER;R26‐tdT* mice were treated with tamoxifen two weeks prior to anastomosis surgery, and FA samples were collected for analysis 1.5 to 3 months post‐operation (**Figure** **2**A). Whole‐mount imaging identified the anastomosis suture site in the injured FA (Figure 2B). The injured FA samples were divided into four zones based on their proximity to the suture site and their post‐injury morphological characteristics (Figure 2C). Hematoxylin and eosin (H&E) staining revealed that Zone 1 (distal to the heart) and Zone 4 (proximal to the heart) exhibited relatively normal physiological phenotypes, albeit with varying diameters. Zone 2, which encompassed the suture site, showed abnormal morphology characterized by thickening of the tunica media and adventitia. Zone 3 also displayed altered morphology, marked by an increased number of adventitial cells and disorganization of SMCs (Figure 2D). Immunostaining for tdT and SMA on FA sections showed that tdT expression colocalized with SMA specifically in Zone 2 (Figure 2E), suggesting that Gli1⁺ ASCs differentiated into SMCs in this region. In contrast, SMA⁺tdT⁺ cells were not observed in the other three zones (Figure 2E) or in sham‐operated FA (Figure 2F). Since SMA expression can also indicate myofibroblast presence during vascular remodeling,^[^ ^40^ ^]^ we performed additional immunostaining with more specific SMC markers—CNN1, SM22, and SM‐MHC—on FA sections. We found that a subset of tdT⁺ cells co‐expressed these SMC markers (Figure 2G–I). These results demonstrate that Gli1⁺ ASCs give rise to SMCs during the pathological remodeling process following anastomosis surgery (Figure 2J).

Under the same vascular anastomosis injury, Sca1⁺Pdgfra⁺ cells were found to contribute to the formation of SMCs.^[^ ^7^ ^]^ To discriminate between these two ASC populations, single‐cell RNA sequencing (scRNA‐seq) was conducted under homeostatic conditions using a *Gli1‐CreER;R26‐tdT* genetic lineage‐tracing mouse model (Figure S3A, Supporting Information). Initial clustering of captured cells based on canonical marker genes identified eight major cell populations (Figure S3B,C, Supporting Information). Subsequent analysis focused on fibroblast and SMC subpopulations (Figure S3D,E, Supporting Information). Stratification of the fibroblast compartment by Gli1, Sca1, and Pdgfra expression delineated three distinct subsets: Gli1⁺Sca1^−^ ASCs, Gli1^−^Sca1⁺ ASCs, and double‐positive Gli1⁺Sca1⁺ ASCs. Differential expression gene (DEG) analysis identified 185 genes that varied significantly among these subpopulations, underscoring considerable transcriptional heterogeneity (Figure S3F, Supporting Information). Gene Ontology (GO) enrichment analysis of these DEGs of three fibroblast subpopulations highlighted divergent involvement in biological processes such as extracellular matrix organization, cell adhesion, cell migration, and calcium ion transport (Figure S3H, Supporting Information). KEGG pathway analysis further revealed marked variation across subtypes in the PI3K–Akt, Rap1, Ras, and Hedgehog signaling pathways (Figure S3I, Supporting Information). Assessment of signaling modules demonstrated that negative regulation of PI3K–Akt signaling, and cell adhesion signaling were significantly downregulated in Gli1⁺ ASCs relative to Sca1⁺Pdgfra⁺ cells. In contrast, Hedgehog, calcium transport signaling and Kras‐DN signaling were upregulated in Gli1⁺ ASCs (Figure S3G, Supporting Information).

Cell‐cycle distribution analysis revealed that Gli1⁺ ASCs harbored a slightly elevated proportion of cells in G_2_/M phase compare to the Sca1⁺ cell population (Figure S3J, Supporting Information). Further assessment of stemness using the CytoTRACE algorithm indicated that Gli1⁺ ASCs retain progenitor‐like potential, albeit to a lesser extent than Sca1^+^ Pdgfra^+^ cells (Figure S3K, Supporting Information). Consistently, Gli1⁺ ASCs exhibited robust expression of canonical mesenchymal stem cell (MSC) markers, including Cd34, Cd44, and Eng, along with the proliferation‐associated gene Pcna and stemness‐related transcription factors Myc and Klf4 (Figure S3L, Supporting Information). These transcriptional features collectively support the classification of Gli1⁺ ASCs as a stem‐like population. Together, these results highlight distinct transcriptional, functional, and signaling programs between Gli1⁺Sca1^−^ and Gli1^−^Sca1⁺ ASCs, underscoring the molecular heterogeneity that exists among adventitial stem cell subsets under homeostatic conditions.

To elucidate the mechanisms underlying Gli1⁺ ASC migration and their contribution to smooth muscle cell (SMC) regeneration, we performed an in‐depth analysis of our single‐cell RNA sequencing (scRNA‐seq) data. Comparative analysis between Gli1⁺ ASCs and Sca1⁺Pdgfra⁺ cells revealed markedly reduced cell adhesion module scores and elevated Ca^2^⁺ transport activity in Gli1⁺ ASCs (Figure S3H, Supporting Information), feature consistent with a heighted potential for participation in vascular repair and remodeling post‐injury.

To test this hypothesis, we performed scRNA‐seq on vascular tissue from *Gli1‐CreER;R26‐tdT* mice following injury‐induced vascular damage and repair. Pseudotime trajectory analysis was performed on the tdT⁺ cell populations, using tdT⁺Gli1⁺ fibroblasts as the root state to reconstruct the continuous trajectory of fibroblast‐to‐SMC differentiation. (Figure S4A–C, Supporting Information). This analysis identified two distinct transitional cell populations—designated SMC1 and SMC2—representing intermediate states, enriched for specific gene expression modules (Figures S3D and S4D, Supporting Information).

Gene Ontology (GO) and KEGG enrichment analyses showed that the SMC1‐associated module was enriched for biological processes related to GTPase regulation, muscle cell differentiation, vascular development, cell morphogenesis, and mesenchymal cell migration (Figure S4E, Supporting Information). In the SMC2 state, key signaling pathways such as MAPK, calcium signaling, cGMP‐PKG, Ras, Rap1, and cell adhesion were implicated as central regulators of the trans‐differentiation process (Figure S4F,G, Supporting Information).

Trajectory‐level scoring of signaling pathways revealed that cGMP signaling, Ca^2^⁺ transport, integrin, and Rap1 pathways were markedly activated during the Gli1⁺ ASC‐to‐SMC differentiation process, accompanied by a moderate engagement of the MAPK pathway, whereas Hedgehog activity remained largely stable with the Gli1⁺ ASCs‐to‐SMCs differentiation trajectory (Figure S4H, Supporting Information). Although Gli1⁺ ASCs exhibited higher basal Hedgehog signaling relative to Sca1⁺Pdgfra⁺ cells (Figure S3H, Supporting Information). Collectively, these findings suggest that Hedgehog signaling plays a permissive rather than instructive role in Gli1⁺ ASC trans‐differentiation, with the transition from Gli1⁺ ASCs to SMCs being primarily orchestrated by other signaling cascades (Figure S4H, Supporting Information).

In the *Gli1‐CreER;R26‐tdT* mice, Gli1⁺ ASCs were genetically labeled with tdT, enabling in vivo tracking of their differentiation into SMCs. This labeling strategy allowed clear distinction between pre‐existing SMCs (tdT^−^ SMCs) and newly differentiated SMCs originating from Gli1⁺ ASCs (tdT⁺ SMCs). Differential gene expression analysis comparing tdT⁺ and tdT^−^ SMCs revealed 44 differentially expressed genes (Figure S4I, Supporting Information), which were primarily associated with biological processes such as smooth muscle differentiation, contractile function, and ion transport (Figure S4J, Supporting Information).

Then, we evaluated the structural and the functional properties of tdT⁺ SMCs derived from Gli1⁺ ASCs. The tdT⁺ SMCs exhibited markedly lower mRNA expression levels of canonical contractile markers—including Acta2, Tagln, and Myh11—than tdT^−^ SMCs. The functional markers associated with cytoskeletal organization (Vcl, Des), contractile regulation (Myl9, Cnn1), myosin phosphorylation (Cald1, Mylk), and Ca^2^⁺ signaling (Cacna1c, Ryr2, Itpr1) was also reduced in tdT⁺ SMCs compared with native tdT^−^ SMCs (Figure S4K, Supporting Information). Collectively, these results demonstrate that SMCs originating from Gli1⁺ ASCs have the structural and functional phenotypes of SMCs but are not in a fully mature state relative to resident vascular smooth muscle cells during vascular remodeling and regeneration.

### 2.3 Gli1⁺ ASCs Do Not Contribute to SMCs Following Femoral Artery Wire Injury

We next investigated whether Gli1⁺ ASCs would differentiate into SMCs in response to a less severe acute vascular injury—specifically, wire injury. We utilized *Gli1‐CreER;R26‐tdT* mice, administering tamoxifen to induce genetic labeling followed by linear wire injury after a 2‐week interval. Femoral arteries were harvested from both sham and injury groups between 2.5 to 4.5 months post‐injury for histological sectioning and immunofluorescence analysis (Figure S7A,B, Supporting Information). Hematoxylin and eosin (H&E) staining demonstrated substantial intimal and medial thickening at the injury site, confirming successful model establishment (Figure S7C, Supporting Information). Immunofluorescence co‐staining for SMC markers (SMA, CNN1, and SM22) and tdT on femoral artery sections revealed robust SMC proliferation at the injury site; however, virtually no tdT‐positive SMCs were detected (Figure S7D,E, Supporting Information). Examination of elastic lamina morphology revealed a continuous and intact medial elastin layer in sham specimens, where the external elastic lamina clearly delineated the adventitial‐medial boundary. In injured vessels, despite pronounced intimal and medial thickening, the elastin structure adjacent to the adventitia remained largely continuous and intact (Figure S7F,G, Supporting Information). Based on these findings, we hypothesized that the structural integrity of the elastin layers likely restricts the migration and subsequent differentiation of Gli1⁺ ASCs into SMCs within the media.

### 2.4 tdT^+^ Cells Minimally Contribute to SMCs in Atherosclerotic Plaques

**Figure 3:**
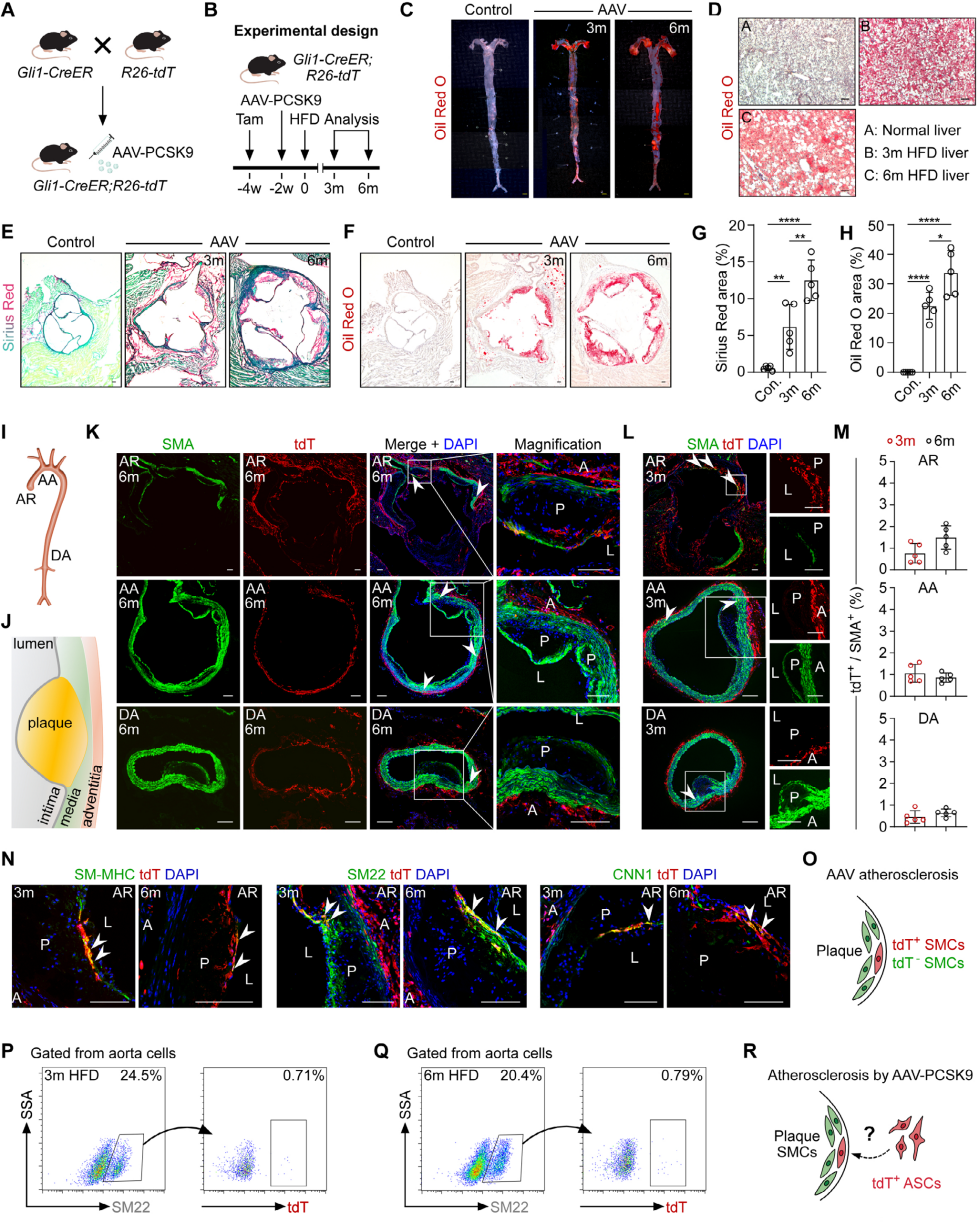
tdT^+^ cells contributed to a minor subset of SMCs in PCSK9‐induced atherosclerosis. A) Schematic diagram of injection of AAV‐PCSK9 into *Gli1‐CreER; R26‐tdT* mice. B) A schematic showing the experimental design. C) Oil Red O staining of whole mounts from control mice and AAV‐PCSK9 (AAV)‐injected mouse aortae after 3 and 6 months of HFD feeding. D) Oil Red O staining of liver sections from the control and AAV‐injected mice. E‐H) Sirius red and Oil Red O staining on aortic root sections from the control and AAV‐injected mice, respectively. Statistics results of the Sirius red staining (G) and Oil Red O staining (H). Data are mean±SD; *n* = 5; *p<0.05; **p<0.01; ****p<0.0001. I,J) A cartoon showing different regions of the aorta (I) and the position of atherosclerotic plaques (J). K–M) Immunostaining for SMA and tdT on cryosections of AR, AA, and DA from the AAV‐injected mice at 6 months (K) and 3 months (L) after HFD feeding. M shows the quantification of the percentage of SMA^+^ cells expressing tdT, *n* = 5. N,O) Immunostaining for tdT and SM‐MHC, SM22, or CNN1 on aortic sections with atherosclerotic plaques from AAV‐injected mice. O shows a cartoon diagram. P,Q) Flow cytometric analysis of AAV‐injected mice after 3 months and 6 months of HFD feeding. R) A cartoon showing the contribution of tdT^+^ ASCs to SMCs in atherosclerotic plaques. White and black scale bars: 100 µm, yellow scale bars: 1000 µm.

To assess whether Gli1⁺ ASCs contribute to SMC formation in atherosclerotic plaques, we employed a mouse model of atherosclerosis using an adeno‐associated virus vector encoding PCSK9 (AAV‐PCSK9), as previously described.^[^ ^41^, ^42^ ^]^ PCSK9 is a liver‐secreted enzyme that binds to the low‐density lipoprotein receptor (LDLR) and induces its degradation. This reduction in LDLR levels leads to elevated circulating cholesterol and lipoprotein levels, which are key drivers of atherosclerotic plaque development.^[^ ^43^, ^44^, ^45^ ^]^ To induce atherosclerosis, AAV‐PCSK9 was administered to 8‐ to 12‐week‐old *Gli1‐CreER;R26‐tdT* mice two weeks after tamoxifen induction (**Figure** **3**A,B). The mice were then maintained on a high‐fat diet (HFD) for either 3 months (short‐term) or 6 months (long‐term). Whole‐mount imaging of aortas stained with Oil Red O revealed lipid accumulation in the atheroma of HFD‐fed mice compared with controls (Figure 3C), demonstrating pronounced atherosclerotic lesion formation. Oil Red O staining of liver sections from AAV‐PCSK9‐treated mice also revealed substantial lipid deposits (Figure 3D). Furthermore, Sirius Red (Figure 3E,G) and Oil Red O (Figure 3F,H; Figure S5B, Supporting Information) staining of aortic sections from these mice confirmed extensive atherosclerotic plaque formation. Collectively, these results demonstrate the successful establishment of an atherosclerosis model in mice via AAV‐PCSK9 injection.

We next investigated the fate of Gli1⁺ ASCs within atherosclerotic plaques across the aortic root (AR), aortic arch (AA), and descending aorta (DA) (Figure 3I). Immunostaining for SMA and tdT on aortic sections from mice fed an HFD for 3 or 6 months revealed sparsely distributed tdT⁺SMA⁺ cells in the AR plaques (Figure 3J,K). Notably, Gli1⁺ ASCs were located predominantly in the adventitia of the AR, AA, and DA, with only minimal tdT⁺ cells detected in the media (Figure 3K,L). Quantitative analysis showed that ≈1% of SMA⁺ cells were tdT‐positive in the AR, AA, and DA of mice fed an HFD for 3 or 6 months (Figure 3M). Immunostaining for SM22, SM‐MHC, and CNN1 on aortic sections confirmed the presence of sparse tdT⁺ SMCs within AR plaques, but hardly any within AA or DA plaques (Figure 3N,O). Of note, tdT⁺ SMCs were detected in the media of AA and DA sections (Figure S5C–E, Supporting Information). Additionally, flow cytometric analysis revealed that 0.71% and 0.79% of SMCs were tdT‐positive in aortas from mice fed an HFD for 3 and 6 months, respectively (Figure 3P,Q). It remained unclear whether these tdT⁺ SMCs were derived from the differentiation of labeled Gli1⁺ ASCs or from the expansion of pre‐existing tdT⁺ SMCs that were ectopically labeled by Gli1‐CreER (Figure 3R). Given the absence of ectopic SMC labeling in the femoral artery (FA) by Gli1‐CreER (Figure S2G, Supporting Information), we examined atherosclerotic plaques in the FA (Figure S5F, Supporting Information). Immunostaining for tdT and SMA on atherosclerotic FA sections revealed no tdT⁺ SMCs in the plaques or media (Figure S5G,H, Supporting Information). These findings highlight a significant concern regarding the ectopic labeling of SMCs by Gli1‐CreER, which complicates the interpretation of lineage‐tracing data in the context of atherosclerosis (Figure 3R).

**Figure 4:**
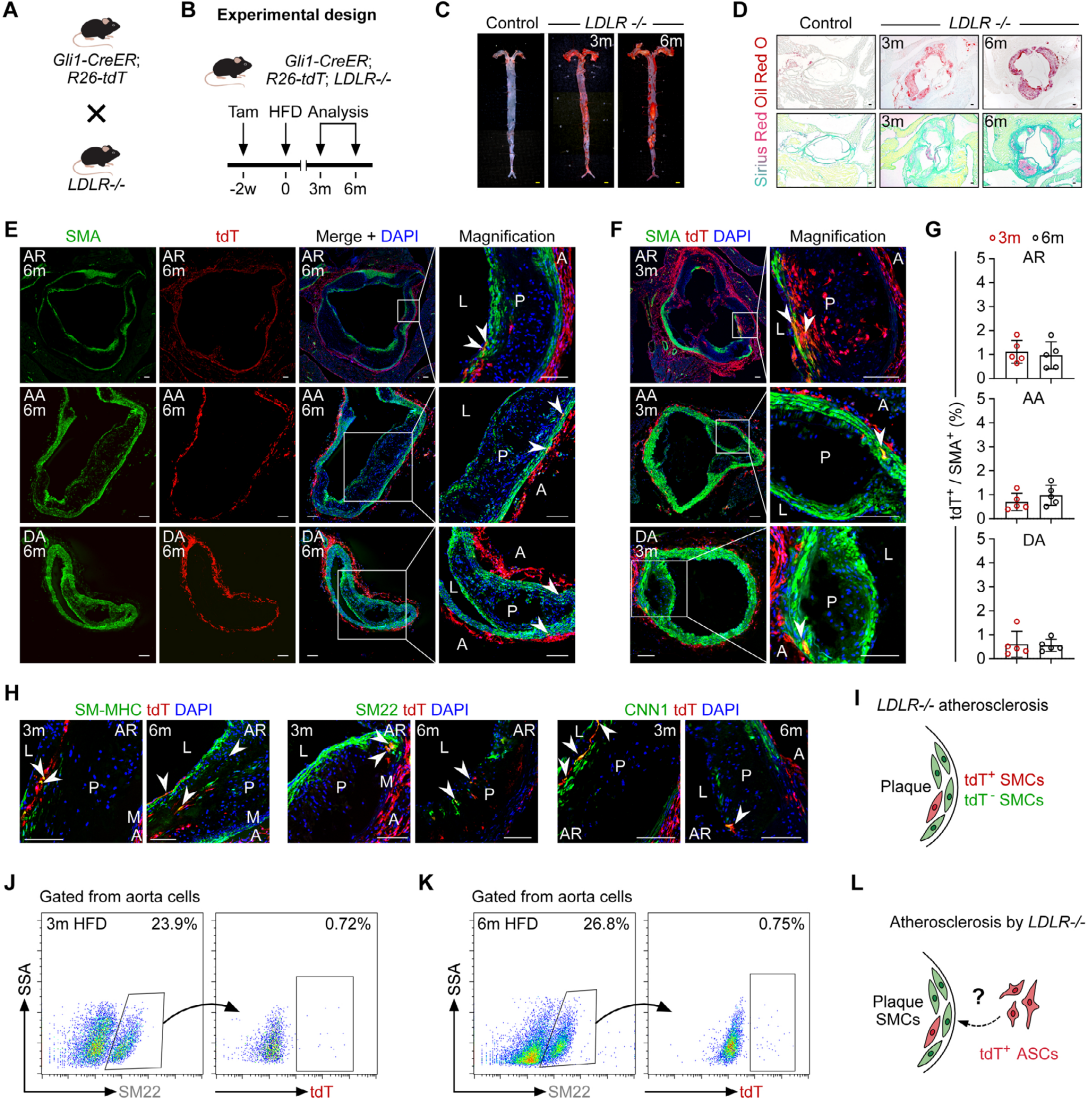
tdT^+^ cells contribute a minor subset of SMCs in *LDLR* ^–/–^ mice. A) Schematic diagram of mice crossing. B) A schematic showing the experimental design. C) Oil Red O staining of the aortas of control mice and *LDLR* ^–/–^ mice fed with an HFD for 3 and 6 months. D) Oil Red O and Sirius red staining results for aortic root sections from control and *LDLR* ^–/–^ mice. E‒G) Immunostaining results using SMA and tdT on cryosections of AR, AA and DA from *Gli1‐CreER;R26‐tdT;LDLR* ^–/–^ mice at 6 (E) and 3 (F) months after HFD feeding. Quantification of the percentage of SMA^+^ cells expressing tdT is shown in G, *n* = 5. H,I) Immunostaining for tdT and SM‐MHC, SM22, or CNN1 on aortic sections with atherosclerotic plaques from *Gli1‐CreER;R26‐tdT;LDLR* ^–/–^ mice. I showing a cartoon diagram. J,K) Flow cytometric analysis of the percentage of SMCs expressing tdT after 3 and 6 months of HFD feeding. L) A cartoon showing the contribution of tdT^+^ ASCs to SMCs in atherosclerotic plaques. White and black scale bars: 100 µm, yellow scale bars: 1000 µm.

To examine the fate of Gli1⁺ ASCs in an alternative atherosclerosis model, we crossed *Gli1‐CreER;R26‐tdT* mice with LDLR‐knockout (*LDLR* ^−^/^−^) mice to generate *Gli1‐CreER;R26‐tdT;LDL* *R* ^−^/^−^ triple‐transgenic mice (**Figure** **4**A). Following tamoxifen treatment, these mice were placed on a high‐fat diet (HFD) from 3 to 6 months of age (Figure 4B). Oil Red O staining of aortic tissues collected after 3 and 6 months of HFD feeding revealed significant atherosclerotic lesions in *LDLR* ^−^/^−^ mice compared with controls (Figure 4C). Further histological analysis of aortic root (AR) sections using Sirius Red and Oil Red O staining confirmed the presence of fibrosis and lipid accumulation, respectively. Oil Red O staining of the aortic arch (AA) and descending aorta (DA) from HFD‐fed mice also showed distinct plaque signals (Figure S6B, Supporting Information), verifying successful atherosclerosis induction in the transgenic mice. Immunostaining for SMA and tdT on aortic sections from HFD‐fed mice revealed sparse tdT⁺SMA⁺ cells in the atherosclerotic plaques of the AR and in the medial layers of the AA and DA (Figure 4E,F). Quantitative analysis showed that 0.5%–1% of SMA⁺ cells were tdT‐positive in the AR, AA, and DA after 3 or 6 months of HFD feeding (Figure 4G). Immunostaining for SM22, CNN1, and SM‐MHC on these sections identified a small population of tdT⁺ SMCs within the AR plaques (Figure 4H,I) and in the medial layers of the AA and DA (Figure S6C–E, Supporting Information). Flow cytometric analysis of cells isolated from the aortas of mice fed an HFD for 3 or 6 months showed that a small proportion (≈0.7%) of SM22⁺ cells were tdT‐positive (Figure 4J,K). Given the absence of ectopic SMC labeling in the femoral artery (FA) by Gli1‐CreER (Figure S2G, Supporting Information), we examined atherosclerotic plaques in the FA (Figure S6F, Supporting Information). Immunostaining for tdT and SMA detected no tdT⁺SMA⁺ cells in the atherosclerotic plaques or medial layers of the FA (Figure S6G,H, Supporting Information). Due to the ectopic labeling of pre‐existing SMCs in the aorta by Gli1‐CreER, it remains unclear whether Gli1⁺ ASCs contribute to SMCs in aortic atherosclerotic plaques (Figure 4L).

### 2.5 Gli1⁺ ASCs Exhibit Minimal Contribution to SMCs in Coronary Artery Atherosclerotic Plaques

Although *LDLR* ^−^/^−^ mice show low susceptibility to spontaneous atherosclerotic plaque formation in coronary arteries, we conducted a series of experimental approaches to investigate this context. First, we analyzed *Gli1‐CreER;R26‐tdT* mice under homeostatic conditions 2–6 months after tamoxifen induction. This long‐term lineage tracing revealed that Gli1‐CreER specifically labeled adventitial cells in coronary arteries without marking medial SMCs (Figure S7H, Supporting Information). Based on this baseline characterization, we then examined cardiac tissues from *Gli1‐CreER;R26‐tdT* mice that received tamoxifen induction, followed by AAV‐PCSK9 injection and 8–9 months of high‐fat diet feeding. Although no plaque formation was observed in the distal coronary arteries, distinct plaques were detected at the coronary artery root (Figure S7I, Supporting Information). Subsequent immunostaining with SMC‐specific markers on sections from this region demonstrated that Gli1⁺ ASCs in the coronary adventitia rarely differentiated into SMCs (Figure S7J–N, Supporting Information).

### 2.6 Specific Labeling of Gli1⁺ ASCs Using a Dual‐Recombinase‐Mediated Genetic System

**Figure 5:**
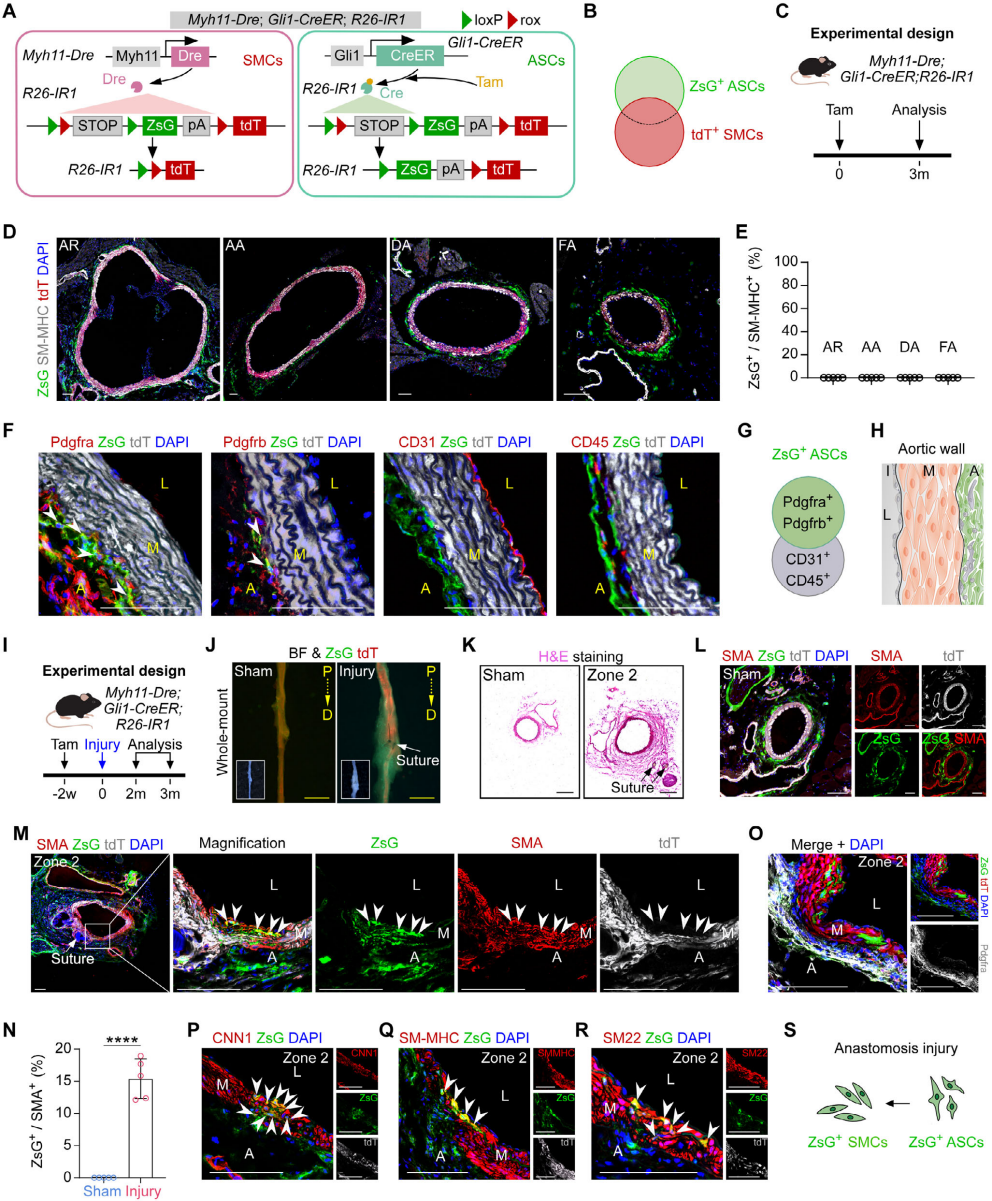
Generation of a dual recombinase‐mediated lineage tracing system to label Gli1^+^ ASCs. A) Schematic diagram of the working principle of dual recombinase‐mediated lineage tracing system to distinctly trace SMCs and Gli1^+^ ASCs. B) A Venn diagram indicating the mutually exclusive labeling by dual recombination. C) A schematic showing the experimental design. D,E) Immunostaining for SM‐MHC, tdT, and ZsG on aortic sections from *Myh11‐Dre;Gli1‐CreER;R26‐IR1* mice treated with Tam. Quantification of the percentage of SM‐MHC^+^ cells expressing tdT is shown in E, *n* = 5. F) Immunostaining for Pdgfra, Pdgfrb, CD31, CD45, tdT, and ZsG on aortic sections. G,H) Cartoons showing that ZsG^+^ ASCs in the vascular adventitia are Pdgfra or Pdgfrb positive, but CD31 or CD45 negative. I) A schematic showing the experimental design. J) Whole‐mount bright‐field and epifluorescence results of sham FA and injury FA. P: proximal, D: distal. K) H&E staining results of cryosections from the sham and injury Zone 2 FA groups. L‐N) Immunostaining for SMA, ZsG, and tdT on FA sham and injury sections. Quantification of the percentage of SMA^+^ cells expressing ZsG is shown in N. Data are mean±SD; *n* = 5; ****p<0.0001. O–R) Immunostaining for ZsG and Pdgfra (O), CNN1 (P), SM‐MHC (Q), or SM22 (R) on FA sections after injury. S) A cartoon showing that the ZsG^+^ ASCs contributed to SMCs under vascular anastomosis injury. White and black scale bars: 100 µm, yellow scale bars: 1000 µm.

To enhance the specificity of lineage tracing for studying Gli1⁺ ASCs, we employed a dual‐recombinase system to address the nonspecific labeling issues associated with single‐recombinase systems.^[^ ^46^ ^]^ Our lineage tracing data indicated that Gli1‐CreER inadvertently labels a minor subset of SMCs in the AR, AA, and DA under normal conditions (Figure S2C,G, Supporting Information). To overcome this limitation, we introduced an additional recombinase (Dre) alongside the conventional Cre‐loxP system. We generated triple‐transgenic mice by crossing *Myh11‐Dre* and *R26‐IR1* mice with *Gli1‐CreER* mice, yielding the *Myh11‐Dre;Gli1‐CreER;R26‐IR1* genotype (**Figure** **5**A). The *R26‐IR1* reporter allele was designed to express two distinct fluorescent proteins—ZsGreen (ZsG) and tdTomato (tdT)—which are activated selectively through Cre‐loxP and Dre‐rox recombination, respectively. In this system, Myh11‐Dre labels SMCs with tdT via Dre‐rox recombination, simultaneously excising the ZsG cassette from the R26‐IR1 allele. Following tamoxifen induction, Gli1‐CreER activates ZsG expression in Gli1⁺ ASCs through Cre‐loxP recombination. This dual‐recombinase configuration ensures that any SMCs pre‐labeled with tdT(and thus lacking the ZsG cassette) cannot be erroneously labeled with ZsG by Gli1‐CreER, even if they express Gli1 (Figure 5A,B). This design effectively prevents mislabeling and enables clear distinction between Gli1⁺ ASCs and SMCs, thereby improving the specificity and accuracy of lineage tracing.

To verify the feasibility of this dual recombinase‐mediated lineage tracing strategy, we first collected the aortic samples of *Gli1‐CreER;R26‐IR1* mice without Tam treatment (Figure S8A, Supporting Information). Immunostaining for ZsG and CNN1 on AR, AA DA, and FA sections revealed no ZsG^+^CNN1^+^ SMCs in the media layer of aortas or other organs, indicating no Cre activity leakiness in the *Gli1‐CreER;R26‐IR1* mice (Figure S8B,C, Supporting Information). Next, we analyzed the aorta and other organs from *Myh11‐Dre;R26‐IR1* mice to characterize SMC labeling (Figure S8D, Supporting Information). Immunostaining for SM‐MHC, CNN1, and tdT on the AR, AA, DA, FA, and other organ sections confirmed efficient labeling of SMCs by tdT (Figure S8E,F, Supporting Information). To characterize the Gli1^+^ ASCs in the dual‐recombinase lineage tracing system, we examined aorta samples from *Myh11‐Dre;Gli1‐CreER;R26‐IR1* triple transgenic mice 3 months post‐Tam treatment (Figure 5C). Immunostaining for ZsG, tdT, and SM‐MHC on AR, AA, DA, and FA sections showed all SMCs were tdT^+^ZsG^–^, indicating that the Myh11‐Dre system specifically and efficiently labeled SMCs (Figure 5D,E). ZsG^+^ cells, marked by Gli1‐CreER, were exclusively located in the vascular adventitia, and there were no ZsG^+^SM‐MHC^+^ cells detected in the medial layer of the aorta (Figure 5D,E) or SMCs from other organs (Figure S5G, Supporting Information). These ZsG^+^ cells expressed Pdgfra and Pdgfrb, but not endothelial (CD31)or hematopoietic cell (CD45) markers (Figure 5F), consistent with the marker expression patterns observed using the single‐recombinase labeling system (Figure 5G). This methodological refinement offers a more reliable and precise tool for studying the Gli1^+^ ASCs in vascular injury, repair, and disease models (Figure 5H).

To evaluate the differentiation potential of Gli1^+^ ASCs during vascular injury, we performed the anastomosis injury on FA of *Myh11‐Dre;Gli1‐CreER;R26‐IR1* triple transgenic mice and analyzed the FA samples 2–3 months post‐injury (Figure 5I). Whole‐mount imaging revealed ZsG and tdT expression in both sham‐operated and injured FA samples (Figure 5J; Figure S9A,B, Supporting Information). H&E staining of these samples demonstrated significant thickening of the vascular media and adventitia in Zone 2 of the injured group, in contrast to the sham‐operated group (Figure 5K; Figure S9C, Supporting Information). Further immunostaining for SMA, ZsG, and tdT on FA sections showed that ZsG^+^ cells, labeled specifically by Gli1‐CreER, were expressing SMA in Zone 2 of the injured FA (Figure 5L–N). We also analyzed Zone 1, Zone 3 and Zone 4 sections of the injury FA samples and confirmed that no ZsG^+^ SMCs appearing (Figure S9D, Supporting Information) which were consistent with the previous data (Figure 2E). Immunostaining for SM‐MHC, CNN1, and SM22 further validated that ZsG^+^ ASCs actively contributed to the formation of SMCs in response to acute vascular injury (Figure 5O–R; Figure S9D, Supporting Information). Overall, these results clearly demonstrate the ability of Gli1^+^ ASCs to differentiate into SMCs, highlighting their significant role in vascular repair and remodeling following injury (Figure 5S).

### 2.7 ZsG^+^ ASCs Do Not Contribute to SMCs in Atherosclerosis

**Figure 6:**
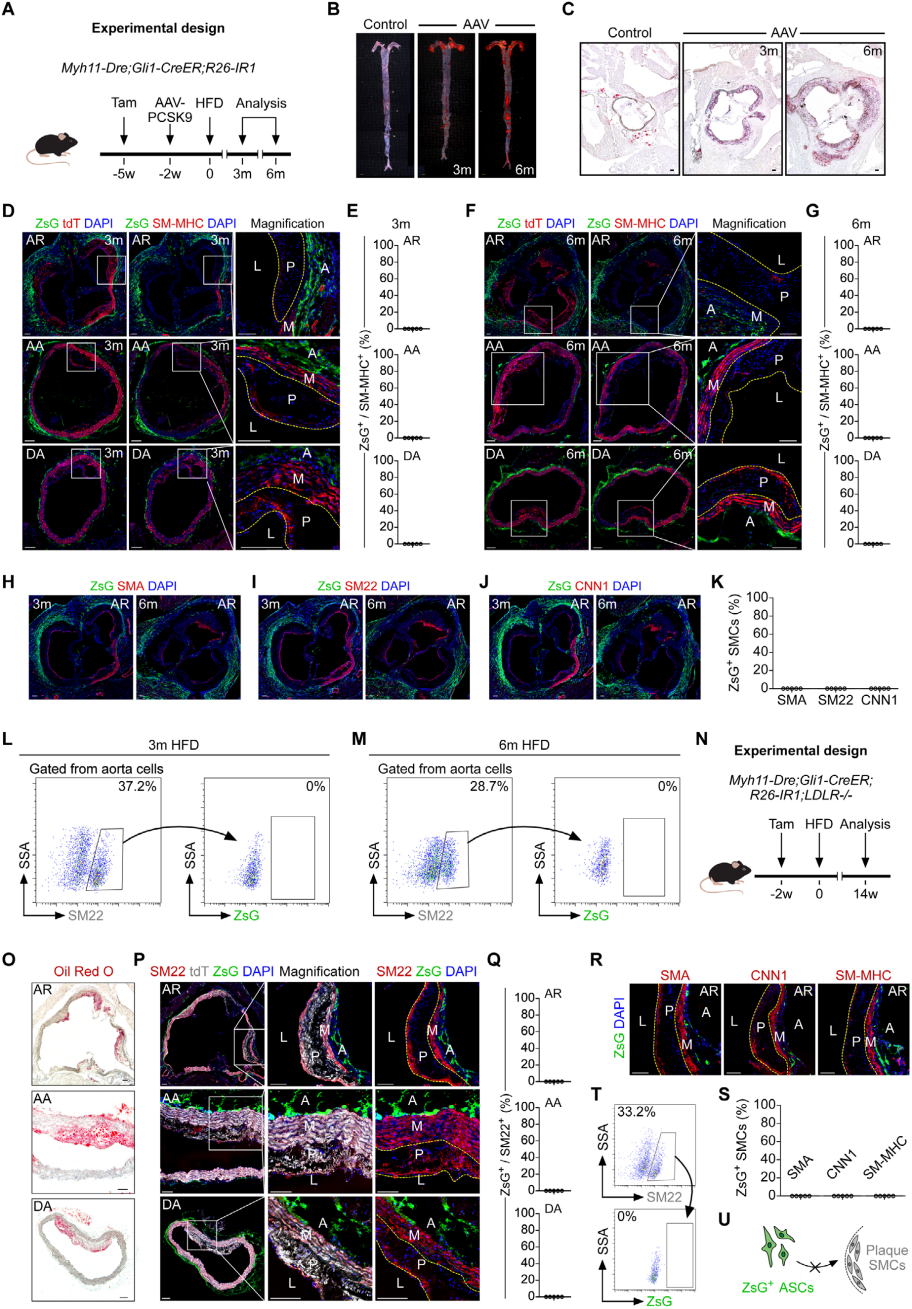
ZsG^+^ ASCs do not contribute to SMCs in atherosclerosis. A) Schematic diagram of the experimental design. B,C) Oil Red O staining of whole‐mount bright‐field images of aortas and sections from the control and AAV‐injected mice after 3 and 6 months of HFD feeding. D–G) Immunostaining for tdT, ZsG, and SM‐MHC on AR, AA, and DA sections from AAV‐injected mice after 3 months (D) and 6 months (F) of HFD feeding. Quantification of the percentage of SM‐MHC^+^ cells expressing ZsG is shown in (E) and (G), *n* = 5. H–K) Immunostaining for ZsG and SMA, SM22, or CNN1 on AR sections. Quantification of the percentage of SMCs expressing ZsG is shown in K, *n* = 5. L,M) Flow cytometric analysis of aortic cells from AAV‐injected mice after 3 or 6 months of HFD feeding. N) A schematic showing the experimental design. O) Oil Red O staining of aortic sections. P–S) Immunostaining for tdT, ZsG, and SM22, SMA, CNN1, or SM‐MHC on aortic sections. Quantification of the percentage of SMCs expressing tdT is shown in Q and S, *n* = 5. T) Flow cytometric analysis of aortic cells. U) A cartoon showing that the labeled ZsG^+^ ASCs did not contribute to SMCs within plaques. White and black scale bars: 100 µm, yellow scale bars: 1000 µm.

To determine whether ZsG⁺ Gli1⁺ ASCs differentiate into SMCs under atherosclerotic conditions, we administered AAV‐PCSK9 to *Myh11‐Dre;Gli1‐CreER;R26‐IR1* triple‐transgenic mice three weeks after tamoxifen induction, followed by a high‐fat diet (HFD) two weeks later to induce atherosclerosis (**Figure** **6**A). Oil Red O staining of aortic samples collected after 3 and 6 months of HFD revealed extensive atherosclerotic plaque formation (Figure 6B,C). Immunostaining for ZsG, tdT, and SM‐MHC on sections of the aortic root (AR), aortic arch (AA), and descending aorta (DA) showed no ZsG⁺ SM‐MHC⁺ SMCs within atherosclerotic plaques or the vascular media after 3 or 6 months of HFD feeding (Figure 6D–G). These ZsG⁺ cells were predominantly localized to the adventitia, consistent with their native niche. Additional immunostaining for CNN1, SM22, and SMA confirmed the absence of ZsG⁺ SMCs in plaques or the medial layer of the vascular wall (Figure 6H–K; Figure S10A–C, Supporting Information). Flow cytometric analysis further supported these observations, as no ZsG⁺ population was detected among isolated SMCs (Figure 6L,M). Taken together, these results indicate that, in contrast to their role following vascular injury, Gli1⁺ ASCs do not give rise to SMCs during atherosclerosis, underscoring their limited contribution to SMC generation in this pathological context.

To further clarify the role of Gli1⁺ ASCs in atherosclerosis, we employed an LDLR‐knockout approach in combination with our dual‐recombinase‐mediated lineage tracing system. We generated *Myh11‐Dre;Gli1‐CreER;R26‐IR1;LDLR* ^−^/^−^ mice and subjected them to a high‐fat diet (HFD) for 14 weeks to induce atherosclerosis (Figure 6N). Oil Red O staining confirmed atherosclerotic plaque formation across multiple regions of the aorta in these quadruple‐transgenic mice (Figure 6O). Immunostaining for SMA, CNN1, SM‐MHC, and SM22, together with ZsG and tdT, on aortic sections revealed that almost all SMCs were tdT‐positive, confirming efficient SMC labeling by the Myh11‐Dre (Figure 6P–S; Figure S7D, Supporting Information). However, no ZsG‐positive SMCs were detected within the plaques or other areas of the aortic tissues (Figure 6P–S; Figure S7D, Supporting Information). Flow cytometric analysis further validated the absence of ZsG‐positive cells within the isolated SMC population (Figure 6T). Collectively, these results, obtained using a dual‐recombinase‐mediated genetic lineage tracing system, demonstrate conclusively that Gli1⁺ ASCs do not give rise to SMCs in atherosclerotic plaques (Figure 6U).

### 2.8 Distinct Cell Fates of Gli1⁺ ASCs in Vascular Homeostasis, Anastomosis Injury, and Atherosclerosis

To delineate differential cell fate transitions in anastomosis injury versus atherosclerosis, we collected femoral artery (FA) specimens from sham‐operated, immediate post‐anastomosis (0‐day), and atherosclerotic groups using tamoxifen‐induced *Myh11‐Dre;Gli1‐CreER;R26‐IR1* mice. These samples were processed for immunohistochemical analysis to evaluate medial elastic lamina integrity (Figure S11A,B, Supporting Information). Comparative analysis revealed that, unlike the continuous elastic lamina in sham controls, Zone 2 of anastomosis‐injured FAs at day 0 exhibited severe elastic lamina fragmentation and disorganization at the injury site. This disruption was accompanied by extensive cell death, as indicated by the absence of tdT⁺ SMC signals. The loss of elastic lamina integrity appears to facilitate Gli1⁺ ASC migration into the media and subsequent fate transition, thereby promoting vascular repair.

In contrast, Zone 3 FAs maintained intact internal and external elastic laminae, with Gli1⁺ ASCs largely confined to the adventitia. In atherosclerotic sections, elastic lamina disruption was localized to plaque‐adjacent regions near the intima or adventitia, coinciding with medial SMC migration toward plaques or the vascular adventitia. Although SMCs in atherosclerosis secrete metalloproteinases ^[^ ^47^, ^48^ ^]^ that partially degrade elastin, the medial elastic lamina largely retained structural continuity, supporting preserved aortic physiological function. Notably, no Gli1⁺ ASC migration from the adventitia to the media or plaques was observed. Based on these findings, we propose that the structural integrity of the medial elastic lamina serves as a physiological gatekeeper that determines whether Gli1⁺ ASCs can enter the media and undergo fate conversion.

## 3 Discussion

In this study, we developed a *Gli1‐CreER;R26‐tdT* single‐recombinase lineage tracing system to efficiently label Gli1^+^ ASCs as tdT^+^ in vivo. Under normal conditions, the labeled Gli1^+^ cells predominantly expressed Pdgfra and Pdgfrb, residing primarily within the vascular adventitia. However, a minor fraction of the tdT signal was detected in the media layer of the aorta, suggesting SMC characteristics in these cells. Detailed analysis of various aortic regions revealed the presence of tdT^+^ cells in the media of the AR, AA, and DA, but not in the FAs. These findings pointed to potential mislabeling issues inherent in the single‐recombinase system for tracing Gli1^+^ ASCs.

**Figure 7:**
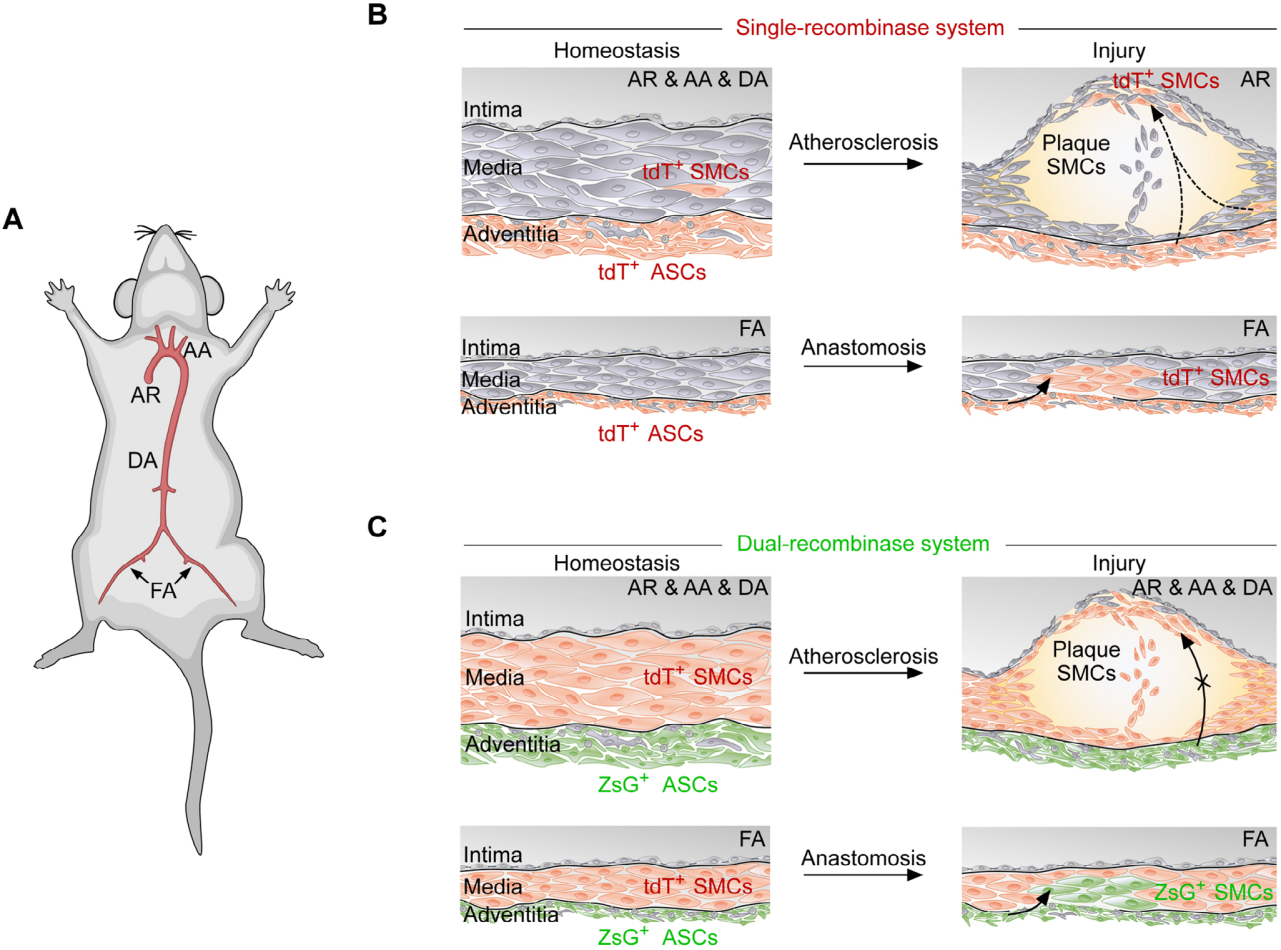
Assessing the contribution of Gli1^+^ ASCs to SMCs by lineage tracing. A) A cartoon showing different segments of the aorta. B,C) The dual‐recombinase system for tracing Gli1^+^ ASCs demonstrated enhanced specificity by eliminating mislabeling events within the vascular media of AR, AA, and DA, compared to the single‐recombinase system. Gli1^+^ ASCs traced by both systems are capable of differentiating into SMCs during vascular anastomosis injury, underscoring their potential role in SMC regeneration in vivo. However, these labeled Gli1^+^ ASCs did not contribute to SMC regeneration within atherosclerotic plaques, highlighting a distinct behavior in different pathological conditions.

To investigate the role of Gli1^+^ ASCs in atherosclerosis, we employed two induction strategies using the lineage tracing system: AAV‐PCSK9 introduction and *LDLR* ^–/–^ models. Our results showed that tdT^+^ cells differentiated into a small subset of SMCs within atherosclerotic plaques. Since tdT^+^ cells expressing SMC markers were present in the vascular media of healthy segments, this raises questions about the origin of the few tdT^+^ SMCs observed in atherosclerotic areas. It remained unclear whether these cells originated from the adventitia stem cells or were simply artifacts of Gli1‐CreER mislabeling (**Figure** **7**A,B). To resolve this, we engineered a dual‐recombinase lineage tracing system, which enhances specificity by restricting the distinct label to the vascular adventitia cells. This advanced system confirmed that while Gli1^+^ ASCs could differentiate into SMCs following acute vascular injury, they do not do so under atherosclerotic conditions (Figure 7C). This finding underscores the variable differentiation capacities of vascular progenitors in response to different types of vascular injury and diseases.

In our dual recombinase‐mediated lineage tracing study, we observed that Gli1^+^ ASCs do not contribute to SMCs within atherosclerotic plaque, which differs from the previous research that reported the conversion of Gli1^+^ ASCs to SMC during neointima formation and their transformation into osteoblast‐like cells during vascular calcification.^[^ ^36^ ^]^ However, Gli1^+^ ASCs labeled by our dual lineage tracing systems can indeed differentiate into SMCs under certain conditions. This finding is consistent with earlier research, which suggests that the differentiation potential of ASCs may vary depending on the specific vascular injury model employed. For example, adventitial Sca1^+^ cells differentiate into SMCs in response to anastomosis injury,^[^ ^7^ ^]^ whereas Wang et al. observed a contrasting role for these cells in the context of atherosclerosis.^[^ ^23^ ^]^ Historically, Li et al. demonstrated that labeled adventitial fibroblasts migrated to the vascular media and contributed to neointima formation following acute balloon injury in rat carotid arteries,^[^ ^49^ ^]^ a phenomenon later reproduced in mice by Lu et al., who found that adventitial Sca1^+^ cells, labeled using *Gli1‐CreER;R26‐YFP*, migrated to the media and expressed SMA under similar injury conditions.^[^ ^37^ ^]^ These studies underscore the fact that vascular injuries involving incisions and resuturing, such as those seen in femoral artery anastomosis or carotid artery balloon injury, disrupt the native three‐layer structure of the vascular wall and compromise elastin integrity, potentially facilitating the migration and subsequent differentiation of ASCs into the media, where the altered microenvironment drives cell fate transition.

In contrast, spontaneous atherosclerosis does not involve incisions, and the elastin layers near the adventitia generally remain intact, likely acting as barriers that physically prevent the migration of adventitial cells into the media. While Hu et al. demonstrated that transplanted Sca1^+^ cells in *ApoE* ^–/–^ mice contributed to media SMCs after vein grafts,^[^ ^21^ ^]^ this also required vascular incisions. Chu et al. suggested that Gli1^+^ cells could differentiate into SMA^+^ cells in non‐muscularized vessels during pulmonary hypertension,^[^ ^50^ ^]^ a condition that disrupts these “barriers” and thus facilitates cell fate transitions. However, SMA expression may also occur in myofibroblasts under disease conditions,^[^ ^51^ ^]^ complicating the assertion that Gli1^+^ cells directly contribute to SMC formation in scenarios without vascular incisions. While Kramann et al. proposed that Gli1^+^ ASCs could differentiate into SMCs during spontaneous atherosclerosis accompanied by nephrectomy in *ApoE* ^–/–^ mice,^[^ ^36^ ^]^ this observation might be confounded by the ectopic labeling of a few SMCs during lineage tracing. Our dual recombinase‐mediated genetic lineage tracing solves the ectopic labeling of SMCs by Gli1‐CreER, and has demonstrated that Gli1^+^ ASCs do not contribute to SMCs during atherosclerosis. Our study presents an intersectional genetic approach for accurately analyzing the cell fate and function of ASCs under various conditions, including arterial homeostasis, acute injury, and atherosclerosis. This refined genetic lineage tracing of ASCs provides compelling evidence of their fate versatility in arterial repair, regeneration, and disease contexts.

## 4 Experimental Section

### Animals

All the mice included in this study were bred on a C57BL/6J/ICR mixed background by the Institutional Animal Care and Use Committee (IACUC) at the Center of Excellence for Molecular Cell Science (CEMCS), Chinese Academy of Sciences. The ethical approval for mice study is obtained from IACUC of CEMCS prior to the research, with the approved number for animal experiments (SIBCB‐S374‐1702‐001‐C1). *Gli1‐CreER* mice were generated by CRISPR/Cas9 technology, and cDNA sequences encoding CreERT2 were inserted via homologous recombination into the Gli1 gene at the ATG locus; this cDNA was subsequently inserted into the WPRE sequence to increase the stability of Gli1 gene RNA transcription. Other mouse lines, including *Rosa26‐tdTomato* (*R26‐tdT*), *Myh11‐Dre*, *LDLR* ^–/–^, and *Rosa26‐IR1* (*R26‐IR1*), were generated as previously described.^[^ ^8^, ^38^, ^46^, ^52^ ^]^ All the experimental mice used in this study were housed in SPF‐class clean animal rooms. The genomic DNA for genotyping was prepared from the tail tissues of 1‐ to 2‐week‐old mice by using lysis buffer containing PK (proteinase K), after which the tissues were lysed overnight in an oven at 55 °C. Then, the mixture was precipitated with isopropanol and washed with 70% ethanol. Lineage tracing mice were generated by the Shanghai Model Organisms Co., Ltd.

### Tamoxifen Treatment

The tamoxifen used in the experiment was dissolved in corn oil at concentration of 20 mg mL^−1^, rotated to dissolve it away from light, and later stored in a 4 °C refrigerator. The experimental mice were treated with tamoxifen at a dose of 0.1–0.2 mg g^−1^ by gavage at 6 to 8 weeks of age after birth. *Gli1‐CreER;R26‐tdT* and *Myh11‐Dre;Gli1‐CreER;R26‐IR1* mice were administered Tam three and five times via oral gavage, respectively.

### Anastomosis Injury

The femoral artery anastomosis model was generated as previously described ^[^ ^7^, ^39^ ^]^ The femoral artery was exposed under a microscope, and blood flow was blocked by ligating the distal and proximal femoral arteries with an 8‐0 medical nylon wire. A small incision was made in the distal femoral artery with spring scissors, and the vascular incision was propped open with a micro‐puncture needle. With the aid of the micro‐puncture needle, a threaded guidewire was introduced into the vessel along the vascular incision, and the proximal ligature was loosened at the same time; the guidewire was introduced into the vessel at a depth of ≈10–15 mm, and maintained for 5 min. After the guidewire was removed and the blood flow to the proximal end of the vessel was re‐established, the vessel was anastomosed with an 11‐0 nylon thread under the microscope, the vessel was fixed by suturing at the two ends of the vessel first, and the middle of the vessel was sutured intermittently. The experimental site was rinsed with saline and sterilized with povidone‐iodine, after which the skin was sutured intermittently with 5–0 cotton spandex thread. Penicillin was injected intramuscularly after surgery. Mice that had completed surgery were placed under a thermostatic heat lamp for rewarming.

### Wire Injury

The femoral artery injury procedure was performed as previously described.^[^ ^7^, ^53^ ^]^ Briefly, mice were anesthetized with 2% isoflurane via an induction chamber, and core body temperature was maintained at 37 °C using a heated pad. During the procedure, respiratory rate was controlled at ≈120–140 breaths per minute with a tidal volume of 0.3–0.5 mL. Surrounding veins and connective tissues were carefully dissected away using microsurgical forceps to isolate the femoral artery. Subsequently, a blunted 31‐gauge needle (diameter: 0.26 mm) was introduced into the isolated femoral artery and advanced 5–10 mm toward the iliac artery, where it remained in place for 3 min. Blood flow was reestablished following ligation of the profunda femoris branch.

### Atherosclerosis Model

Two strategies for disrupting the LDL receptor were used in this study: intraperitoneal injection of AAV2/8‐D377Y‐mPCSK9 and LDLR knockout (*LDLR* ^–/–^). The AAV2/8‐D377Y‐mPCSK9 virus was packaged and purified by Taitool Biotechnologies Company (Shanghai, China). All the experimental mice that received tamoxifen gavage and were washed for at least 2 weeks were injected intraperitoneally with 3–4 × 10^11^ genome copies of the virus per mouse. Mice were fed a high‐fat diet (HFD) 2 weeks after AAV injection. The *LDLR* ^–/–^ mice were fed an HFD 2 weeks after tamoxifen treatment. High‐fat chow was used as a high‐fat (40 kcal%) high‐cholesterol (1.25%) mouse food (Dyets, ASHF3). Mouse aorta samples were collected after HFD feeding for 3 or 6 months. The aortic root, aortic arch, and descending aorta segments were specifically analyzed. On average, 30–40 sections per mouse were collected from each of these aortic regions for analysis and statistical evaluation. Furthermore, the selection of atherosclerotic sections was based on the following criteria: the presence of atherosclerotic plaques on the luminal side of the aorta, along with structural features including smooth muscle cell dedifferentiation and fibrous cap formation.

### Whole‐Mount Microscopy

After the mice were euthanized using CO_2_, the organ tissues were collected, washed with PBS to remove excess blood, immersed in 4% PFA solution for 1 h for fixation, washed three times with PBS for 5 min each, added to PBS and placed in a refrigerator at 4 °C for 30 min. Afterward, the washed organ tissues were placed on a black background plate, and PBS was added so that the liquid surface exceeded the mouse organ tissues; if the mouse organ tissues were not easy to fix, a 0.01 mm diameter insect dissection needle was used to assist the fixation. After the mouse organ tissue was fixed, bright‐field and fluorescence images of the whole organ tissue were captured using a Zeiss stereomicroscope (AxioZoom V16).

### Immunofluorescence Staining

The fixed and washed organ tissues were dehydrated overnight using 30% sucrose and then placed in a tissue embedding box. The optimum cutting temperature (O.C.T., Sakura) was used to adjust the tissues, and the tissues were pre‐embedded at 4 °C for at least 1 h. After that, the samples were frozen at −20 °C. Next, the tissues were cryo‐sectioned or stored at −80 °C. For each block, 10 mm thick cryosections were collected for further immunostaining. Before immunostaining, the cryosections were immersed in PBS twice, once for 5 min each, to adequately remove excess O.C.T from the sections. Then, the sections were incubated closed using blocking buffer (2.5% donkey serum and 0.2% Triton X‐100 dissolved in PBS) for at least 30 min at room temperature and incubated with primary antibody overnight (12–24 h) at 4 °C in the dark. In this study, the following primary antibodies were used: Gli1 (Affinity Biosciences, DF7523, 1:100), SMA (Sigma, F3777, 1:500; Abcam, AB5694, 1:100), tdT (Rockland, 200‐101‐379. 1:1000, Rockland, 600‐401‐379, 1:1000, ChromoTek, 5f8, 1:100), SM22 (Abcam, AB14106, 1:500), CNN1 (Abcam, AB46794, 1:300), SM‐MHC (Abcam, AB224804, 1:300), PECAM‐1 (Ebioscience, 17‐0311‐82, 1:100), CD45 (Ebioscience, 17‐0451‐82, 1:100), Pdgfra (R&D, AF1062, 1:500), Pdgfrb (Ebioscience, 14‐1402‐82, 1:500), Alexa Fluor hydrazides (Invitrogen, A20502, 1:100) and ZsGreen (Clontech, 632474, 1:1000). Fluorescence signals were detected by using Alexa fluorescence‐conjugated secondary antibodies for 30 min at room temperature. The following second antibodies were used: Alexa donkey a‐goat 555 (Invitrogen, A21432, 1:1000), Alexa donkey a‐goat 647 (Invitrogen, A21447, 1:1000), Alexa donkey a‐rabbit 555 (Invitrogen, A31572, 1:1000), Alexa donkey a‐rat 647 (Abcam, AB150155, 1:1000), Alexa donkey a‐rabbit 647 (Invitrogen, A31573, 1:1000). For Gli1 or ZsGreen staining, the sections were incubated with Immpress solution (30 µl 30% H_2_O_2_ in 1 ml PBS) until the bubbles disappeared, and the samples were washed with PBS before they were incubated with blocking buffer. After removing the primary antibody, the sections were incubated with Immpress antibody (Vector Lab, MP‐7401‐50, 1:3) for at least 30 min at room temperature in the dark. It was next used a tyramide signal amplification kit (PerkinElmer, NEL741001KT, 1:1000) for 5–10 min in the dark at room temperature. Because ZsGreen, SMA, SM22, CNN1, and SM‐MHC antibodies were obtained from rabbit sources, for the co‐immunostaining of ZsGreen with a smooth muscle cell antibody, we used double rabbit staining. In this protocol, ZsGreen was stained first as described above. Afterward, the sections were incubated with blocking buffer for 1 h at room temperature, followed by incubation with 1% chrompure rabbit IgG (JIR, 011‐000‐003, 1:100) diluted in PBST (0.2% Triton X‐100) for 1 h at room temperature and washing with PBS for 30 min. Next, the sections were blocked with Fab Fragment Donkey Anti‐rabbit IgG (H+L) (JIR, 711‐007‐003, 1:100) for 1 h at room temperature and washed with PBS for 30 min. Afterward, the sections were incubated with a smooth muscle antibody at 4 °C overnight, and an Alexa Fluor II antibody was used to develop fluorescence signals. Finally, the sections were mounted with a mounting medium containing the nuclear stain DAPI (Thermo Fisher Scientific, 62248, 1:1000). All images were acquired using Olympus confocal microscope (FV3000 and FV4000) and Nikon confocal microscope (Nikon A1).

### Oil Red O Staining

Tissue cryosections were air‐dried and soaked in PBS for 5 min, after which the mixture was changed to fresh PBS for another 5 min to fully remove the O.C.T. around the tissues. Next, the Oil Red O staining solution was prepared. The Oil Red O working solution needs to be reconstituted each time, as this process does not last long. The O.C.T.‐removed tissue cryosections were immersed in 60% isopropanol for 10 min, followed by immersion in Oil Red O staining solution for 15 min (2–3 h if the staining was applied to mouse organs). Afterward, the cryosections were placed in 60% isopropyl alcohol for 20 s until the background of the cryosections appeared clear. The sections were then placed in PBS for 5 min, after which the sections were blocked by adding 2 drops of glycerol sealer to each slide. Oil Red O staining revealed a reddish coloration of the lipids and a blue coloration of the nuclei. Oil red O staining solution was prepared in two steps. The first step involved preparing the stock strain by dissolving 0.5 g of Oil Red O in 100 ml of isopropanol, and the second step involved mixing 30 ml of the stock strain with 20 ml of deionized water, allowing it to stand for 10 min, and then filtering it through filter paper. The Oil Red O dye working solution was used to seal the samples as soon as possible after filtration to avoid prolonged exposure to the air. Since this Oil Red O staining working solution cannot be stored for a long time, it needs to be used within 2 h after preparation.

### Sirius Red Staining

Sirius red staining was performed as previously reported.^[^ ^54^ ^]^ The tissue cryosections were removed, dried, soaked in PBS for 5 min, incubated in fresh PBS, and soaked for another 5 min to fully remove the O.C.T. from the sections. The sections were fixed in 4% PFA for 15 min, rinsed 3 times with PBS, and then immersed in Bouins' solution (9% formaldehyde, 5% acetic acid, and 0.9% picric acid) overnight at room temperature (12–24 h), stained in 0.1% fast green solution (Fisher, F‐99) for 4 min and rinsed 3 times with deionized water. Then, the sections were reacted in 1% acetic acid solution for 1 min, stained in 0.1% Sirius Red solution (Direct Red 80, Sigma, 0–03035) for 10 min, and rinsed 3 times with deionized water. Finally, the slides were dehydrated in a series of 95% ethanol, 100% ethanol, and xylene. All images were captured with an Olympus microscope (Olympus, DP72).

### H&E Staining

H&E staining was performed as previously described.^[^ ^54^ ^]^ After removing the O.C.T. from the PBS, the sections were immersed in hematoxylin A staining solution for 20 min at room temperature and washed with deionized water 3 times. Next, the sections were placed in 1% hydrochloric acid for 1 min, washed with deionized water 3 times, immersed in 1% ammonia for 1 min, washed with eosin for 30 s, and then placed in 95% ethanol for 1 min. Afterward, the sections were incubated in ammonia for 1 min. The residual solution was washed with deionized water, placed in 95% ethanol for 1 min, and stained with eosin‐Y for 10 s, followed by a series of dehydration with ethanol and xylene. Finally, the slides were mounted with neutral balsam after drying. All images were obtained with an Olympus microscope (Olympus, DP72).

### Aortic Cell Isolation and Flow Cytometry

Aortic tissues were collected from the mice perfused with PBS, finely minced and subsequently transferred to a digestion mixture (DMEM; 1.5 mg mL^−1^ collagenase, Type 1 [Gibco, 17100‐017]; 1.5 mg mL^−1^ collagenase, Type 2 [Worthington, LS004177]; 2 U mL^−1^ elastase [Worthington, LS002279]; and 10% fetal bovine serum [Thermo Fisher, 10099141c]). Aortic tissues were incubated in the digestion mixture at 37 °C for 40 min on a shaker at a speed of 120 rpm. After digestion, the cell suspension was filtered through a 70 µm cell strainer and then pelleted by centrifugation at 500 g for 5 min. 1X RBC lysis buffer (eBioscience, 00‐4333‐57) was added for blood cell clearance if necessary. After the cells were washed with PBS, they were centrifuged at 500 g for 5 min, after which the supernatant was discarded. The cell precipitate was resuspended in PBS. The Intracellular Fixation & Permeabilization Buffer Set (Invitrogen, 88‐8824‐00) was used to process the cells before staining. After that, the cells were separately stained with transgelin (SM22) (Santa Cruz, sc‐53932 AF647), CD45‐APC (eBioscience, 47‐0451‐82, 1:400), and CD31‐APC (eBioscience, 17‐0311‐82, 1:40) for 30 min at 4 °C and then washed with PBS. Finally, the cells were resuspended in PBS and analyzed with a Thermo Multiwell Plate Failure Stem Cell Multicolor Flow Cytometry Instrument (Attune NxT). The flow cytometric data were analyzed with FlowJo software.

### RNA Isolation and Quantitative RT‐PCR

Total RNA was isolated from harvested cells or lung tissue using Trizol reagent (Invitrogen, Cat# 15596018) according to the manufacturer's instructions. The extracted RNA was subsequently reverse‐transcribed into complementary DNA (cDNA) using the PrimeScript RT reagent kit (Takara, Cat# RR047A). Quantitative PCR was performed using the SYBR Green qPCR Master Mix (Thermo Fisher Scientific, Cat# 4367659) on a QuantStudio 6 Real‐Time PCR System (Applied Biosystems, Thermo Fisher Scientific). In all qPCR experiments, the mRNA expression level of the housekeeping gene Gapdh was measured as an internal control. To assess the expression of hedgehog signaling‐related genes, the mRNA levels of Smo, Sufu, Ptc1 and Gli1 were analyzed using gene‐specific primers. The nucleotide sequences of the primers used were as follows: Gapdh: forward primer:TTGTCTCCTGCGACTTCAAC, reverse primer: GTCATACCAGGAAATGAGCTTG, Smo: forward primer: GAGCGTAGCTTCCGGGACTA, reverse primer: CTGGGCCGATTCTTGATCTCA, Sufu: forward primer: GGGACTGCACGCCATCTAC, reverse primer: TTGACGATAGCGGTAACCTGG, Ptch1: forward primer: AAAGAACTGCGGCAAGTTTTTG, reverse primer: CTTCTCCTATCTTCTGACGGGT, Gli1: forward primer: CCAAGCCAACTTTATGTCAGGG, reverse primer: AGCCCGCTTCTTTGTTAATTTGA.

### ScRNA‐Seq Data Processing

Femoral artery single‐cell suspensions were processed on the 10x Genomics Chromium Controller using the Chromium Single Cell 3′ Reagent Kits v3, following the manufacturer's instructions. cDNA amplification and library construction were performed according to the standard protocol. Sequencing was carried out on an Illumina NovaSeq 6000 platform (paired‐end, 150 bp, multiplexed) by LC‐Bio Technology Co., Ltd. (Hangzhou, China). Raw sequencing data were processed with Cell Ranger (v9.0.1), including alignment to a customized mouse reference based on the database (Ensembl release 105), alignment filtering, barcode processing, and unique molecular identifier (UMI) quantification. Initial quality control (QC) and downstream analyses were performed in Seurat (v5.3.0). Genes detected in fewer than three cells were removed first. Then we excluded cells with < 200 detected features and subsequently retained high‐quality cells with 1500–8000 detected genes and a mitochondrial read fraction <20%. Putative doublets were identified and removed with scDblFinder (v1.22.0). After QC, 5953 cells from the sham sample and 8104 cells from the injury sample were retained, with a median of 12146 UMIs per cell across the combined dataset.

### ScRNA‐Seq Data Integration

Normalization and variance stabilization were performed on raw UMI counts matrix using SCTransform function with Seurat. Datasets (sham and injury) were integrated with Seurat's SCT‐based canonical correlation analysis (CCA) workflow, including the SelectIntegrationFeatures, PrepSCTIntegration, FindIntegrationAnchors, IntegrateData functions. Principal component analysis (PCA) was computed on the resulting SCT residuals using RunPCA function; the number of informative components was assessed with ElbowPlot function, and 30 principal components (PCs) were used for nonlinear embedding with RunUMAP function. A shared nearest‐neighbor graph was constructed with FindNeighbors, and graph‐based clustering was performed with FindClusters at a resolution of 0.1 to define major populations; subclustering of selected populations used a resolution of 0.3.

### ScRNA‐Seq Data Analysis

Differentially expressed genes (DEGs) analysis employed FindAllMarkers function with parameter set as min.pct = 0.25 and logfc.threshold = 0.25. Functional enrichment analysis of DEGs was conducted using clusterProfiler (v4.16.0) against Gene Ontology (GO) and Kyoto Encyclopedia of Genes and Genomes (KEGG) databases with default settings. Developmental potential and lineage inference combined CytoTRACE2 (cytotrace2_py v1.1.0) and Monocle 3 (v1.4.26). Specifically, in the injury group, CytoTRACE2 scores were computed for tdTomato⁺ cells, and tdTomato⁺Gli1⁺ fibroblasts were specified as root cells for trajectory reconstruction in Monocle 3 (preprocessing, UMAP reduction, principal graph learning, and cell ordering using default parameters). Cell cycle analysis was based on the G2/M and S phase markers using CellCycleScoring function. All the pathway associated genes for module scoring were obtained from the Mouse collections of the Molecular Signatures Database (MSigDB) via the GSEA portal (https://www.gsea‐msigdb.org/gsea/msigdb/mouse/genesets.jsp), including REACTOME_NEGATIVE_REGULATION_OF_THE_PI3K_AKT_NETWORK.v2025.1.Mm.gmt, HALLMARK_KRAS_SIGNALING_DN.v2025.1.Mm.gmt, REACTOME_RAP1_SIGNALLING.v2025.1.Mm.gmt, BIOCARTA_INTEGRIN_PATHWAY.v2025.1.Mm.gmt, GOBP_CELL_CELL_ADHESION_MEDIATED_BY_INTEGRIN.v2025.1.Mm.gmt, GOBP_CALCIUM_ION_TRANSPORT.v2025.1.Mm.gmt, REACTOME_SIGNALING_BY_HEDGEHOG.v2025.1.Mm.gmt, GOBP_CGMP_MEDIATED_SIGNALING.v2025.1.Mm.gmt, BIOCARTA_MAPK_PATHWAY.v2025.1.Mm.gmt.

### Statistical Analysis

All the data were obtained from multiple individual biological samples, as indicated in each Figure legend and are presented as the means ± standard deviation (SD). The “n” in the article represented the number of biological replicates. For statistical comparisons, an unpaired two‐sided Student's t test was performed using Graphpad software for comparing differences between two groups and one‐way ANOVA followed by the Turkey method for over two groups. Significance was accepted when p < 0.05.

## Conflict of Interest

The authors declare no conflict of interest.

## Author Contributions

H.W., X.H. and J.M. contributed equally to this work. H.W., C.N., and B.Z. conceived and designed the project; J.M is responsible for analyzing single‐cell sequencing data. X.H., E.W., Y.L., W.P., Y.X., X.G., and M.C. bred mice, performed mouse experiments, and analyzed the data; L.W., B.Z., and C.N. co‐supervised the study, interpreted data, and revised manuscript.

## Supporting information



Supporting Information

## Data Availability

The raw data for scRNA‐seq in this study have been deposited into the Gene Expression Omnibus database under accession number GSE310863 and are publicly available at https://www.ncbi.nlm.nih.gov/geo/query/acc.cgi?acc=GSE310863.
