# Supplementary material for: Contribution of Gli1+ Adventitial Stem Cells to Smooth Muscle Cells in Atherosclerosis and Vascular Injury
Source: Adv Sci (Weinh). 2025 Dec 3;13(14):e12897. doi: 10.1002/advs.202512897 (PMC12970261; doi:10.1002/advs.202512897)
Supplement: Supplementary file 1 — Supporting Information [file ADVS-13-e12897-s001.pdf]

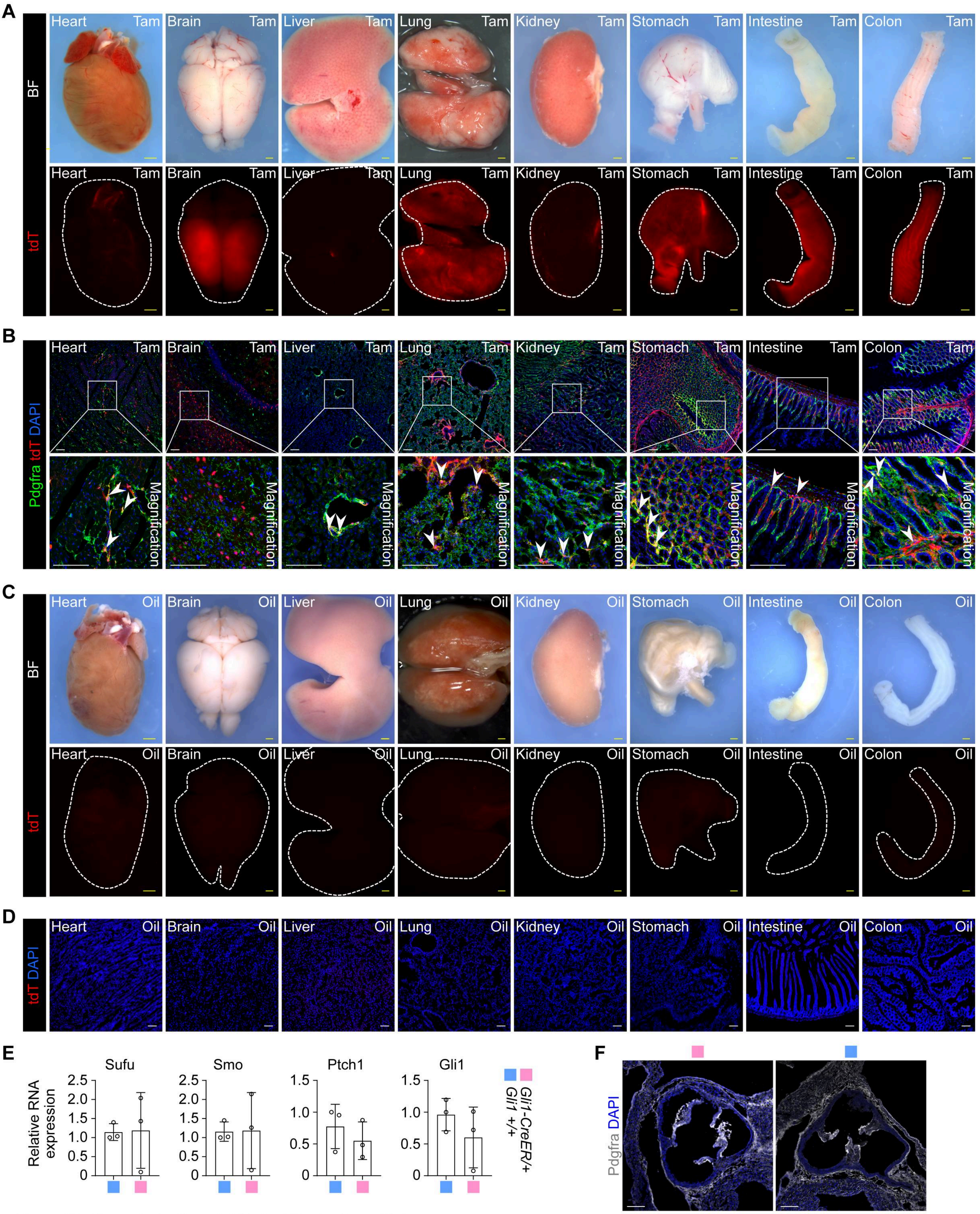

Figure S1. Characterization of *Gli1-CreER* mice in multiple organs.
(A-B) Whole-mount (A) and immunostaining (B) results of multiple organs from *Gli1-CreER;R26-tdT* mice with tam treatment.
(C-D) Whole-mount (C) and immunostaining (D) results of multiple organs from *Gli1-CreER;R26-tdT* mice with oil treatment.
(E) Quantification of the expression of hedgehog signal genes in adventital cells of aorta from 5w-old *Gli1-CreER* heterozygous mice and their wild-type littermates, n=3 respectively.
(F) Immunostaining of aortic root sections from P9 *Gli1+/+* and *Gli1-CreER/+* mice using Pdgfra antibody.
Yellow scale bars: 1000 μm, White scale bars: 100 μm.

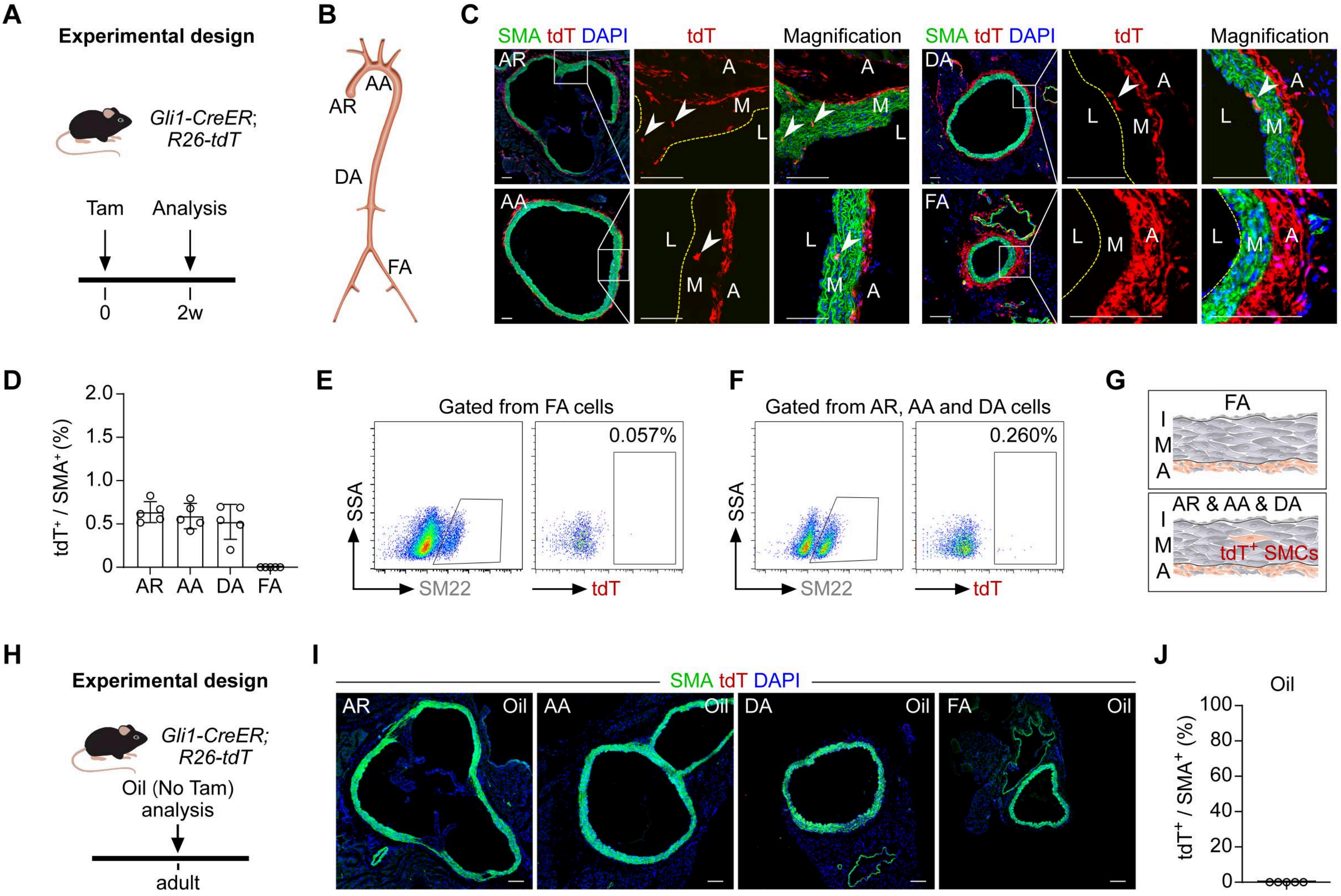

Figure S2: Characterization of different area of aortas of *Gli1-CreER;R26-tdT* mice with Tam or Oil treatment.
(A) Experimental design for tamoxifen (Tam) or Oil treatment and tissue analysis.
(B) Schematic depicting the four vascular segments analyzed: aortic root (AR), aortic arch (AA), descending aorta (DA), and femoral artery (FA).
(C) Representative immunofluorescence images of AR, AA, DA, and FA sections from Tam-treated *Gli1-CreER;R26-tdT* mice, stained for SMA (green) and tdTomato (tdT, red).
(D) Quantification of the percentage of tdTomato-positive (tdT^+) SMCs in each segment from (C), n=5.
(E, F) Flow cytometric analysis of tdT expression in SMC populations isolated from the indicated segments of Tam-treated mice.
(G) Summary diagram illustrating that tdT^+ SMCs were detected in the AR, AA, and DA, but not in the FA.
(H) Experimental design for the Oil-treated control group.
(I) Representative immunofluorescence images of AR, AA, DA, and FA sections from Oil-treated control mice.
(J) Quantification of tdT^+ SMCs from (I), n=5.
White scale bars: 100 μm.

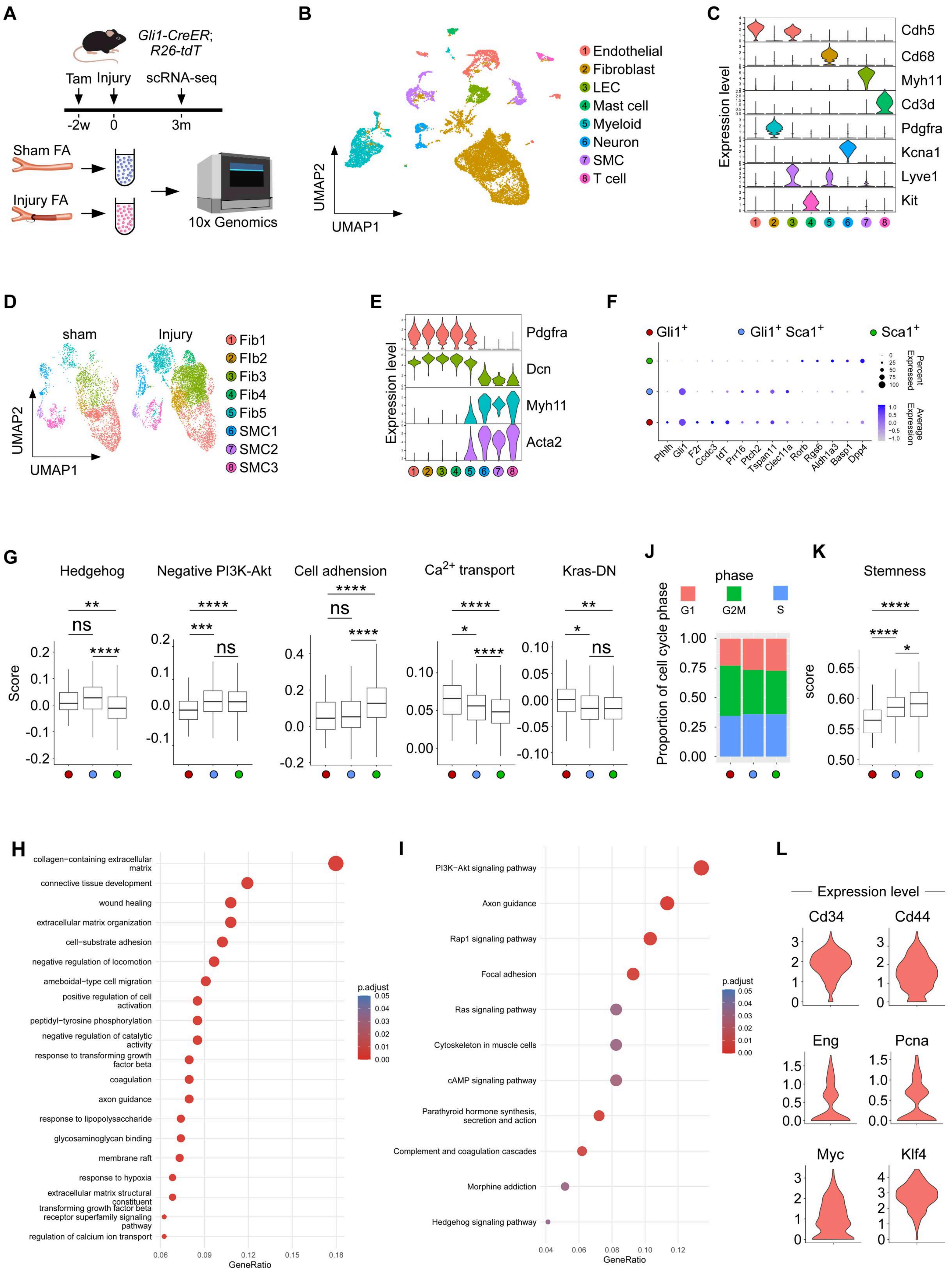

A
Gli1-CreER; R26-tdT
Tam
Injury
scRNA-seq
-2w
0
3m
Sham FA
Injury FA
10x Genomics
B
UMAP1
UMAP2
1 Endothelial
2 Fibroblast
3 LEC
4 Mast cell
5 Myeloid
6 Neuron
7 SMC
8 T cell
C
Expression level
Cdh5
Cd68
Myh11
Cd3d
Pdgfra
Kcna1
Lyve1
Kit
D
sham
Injury
UMAP1
UMAP2
1 Fib1
2 FIb2
3 Fib3
4 Fib4
5 Fib5
6 SMC1
7 SMC2
8 SMC3
E
Expression level
Pdgfra
Dcn
Myh11
Acta2
F
Gli1+
Gli1+ Sca1+
Sca1+
Percent Expressed
Average Expression
Pthlh
Gli1
F2r
Ccdc3
tdT
Prr16
Ptch2
Tspan11
Clec11a
Rorb
Rgs6
Aldh1a3
Basp1
Dpp4
G
Hedgehog
Negative PI3K-Akt
Cell adhesion
Ca2+ transport
Kras-DN
Score
J
phase
G1
G2M
S
Proportion of cell cycle phase
K
Stemness
score
H
collagen−containing extracellular matrix
connective tissue development
wound healing
extracellular matrix organization
cell−substrate adhesion
negative regulation of locomotion
ameboidal−type cell migration
positive regulation of cell activation
peptidyl−tyrosine phosphorylation
negative regulation of catalytic activity
response to transforming growth factor beta
coagulation
axon guidance
response to lipopolysaccharide
glycosaminoglycan binding
membrane raft
response to hypoxia
extracellular matrix structural constituent
transforming growth factor beta receptor superfamily signaling pathway
regulation of calcium ion transport
p.adjust
GeneRatio
I
PI3K−Akt signaling pathway
Axon guidance
Rap1 signaling pathway
Focal adhesion
Ras signaling pathway
Cytoskeleton in muscle cells
cAMP signaling pathway
Parathyroid hormone synthesis, secretion and action
Complement and coagulation cascades
Morphine addiction
Hedgehog signaling pathway
p.adjust
GeneRatio
L
Expression level
Cd34
Cd44
Eng
Pcna
Myc
Klf4

Figure S3. ScRNA-seq analysis of femoral artery cells
(A) The experimental design for preparation of scRNA-seq.
(B) Visualization of unsupervised clustering in a uniform manifold approximation and projection (UMAP) plot of cells isolated from femoral artery (FA) of Tam-treat *Gli1-CreER;R26-tdT* mice after sham or anastomosis injury.
(C) Representative cell markers of each cell clusters.
(D) Visualization of unsupervised clustering in a UMAP plot of fibroblasts and SMCs subpopulations.
(E) Representative Fibroblasts- and SMCs- related markers of each cell clusters from (D).
(F, G) Differential expressed genes (DEG) analysis in (F) and signaling pathway module score assessment in (G) of Gli1$^+$ Sca1$^-$, Gli1$^-$ Sca1$^+$ and Gli1$^+$ Sca1$^+$ cells in sham FA. Signaling pathway module gene sets are from GSEA database. Performed pairwise comparisons between group levels using the pairwise wilcoxon test.
(H, I) The results of Gene Ontology (GO) enrichment analysis and KEGG pathway analysis of DEGs of Gli1$^+$ Sca1$^-$, Gli1$^-$ Sca1$^+$ and Gli1$^+$ Sca1$^+$ cells in sham FA.
(J-K) Comparison of cell cycle phase proportion by CellCycleScoring function in (J) and the stemness state in (K) of Gli1$^+$ Sca1$^-$, Gli1$^-$ Sca1$^+$ and Gli1$^+$ Sca1$^+$ cells by CytoTRACE score.Performed pairwise comparisons between group levels using the pairwise wilcoxon test.
(L) Expression level of stemness related marker genes of Gli1$^+$ ASCs in sham FA.

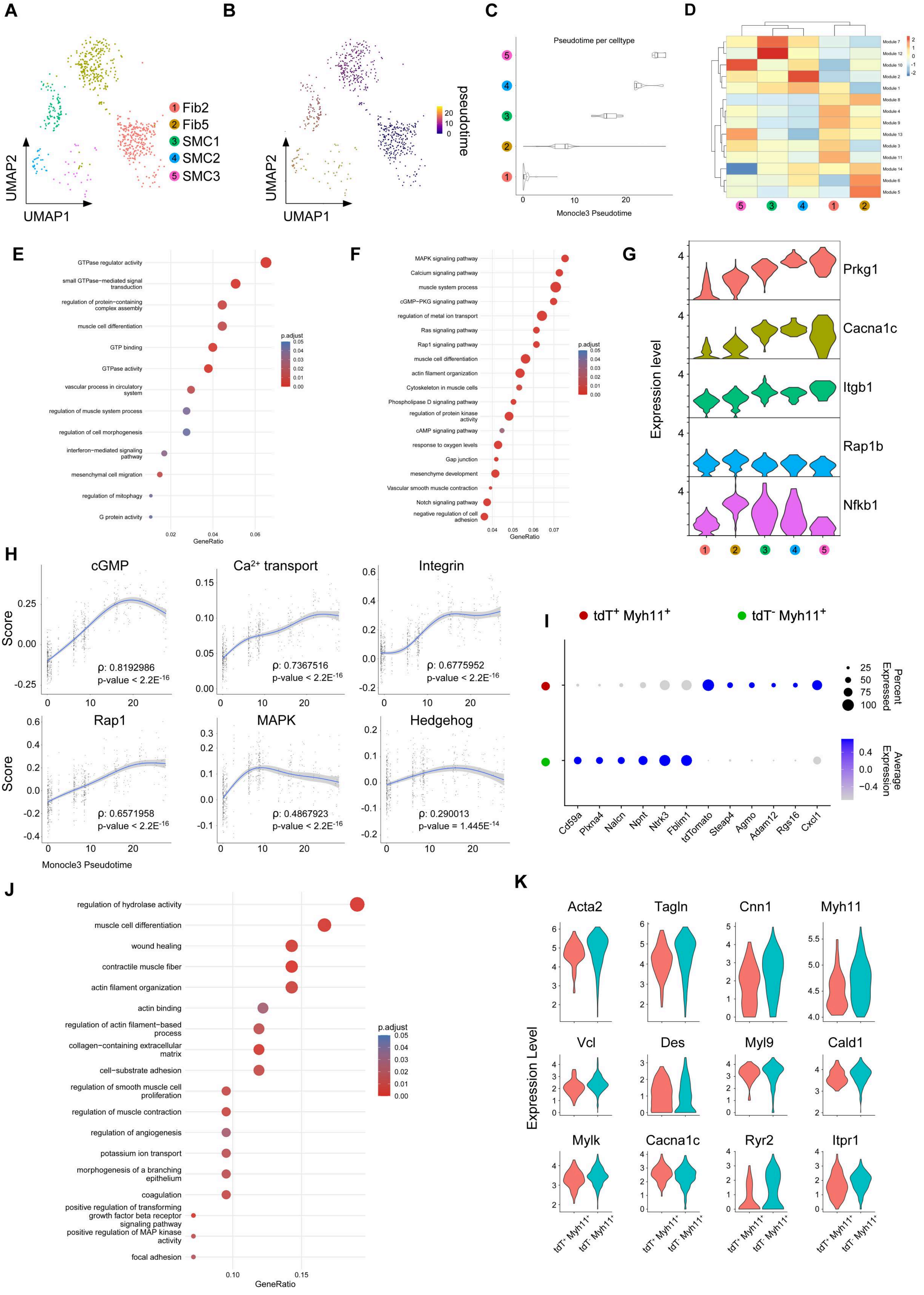
A
B
C
D
UMAP2
UMAP1
1 Fib2
2 Fib5
3 SMC1
4 SMC2
5 SMC3
pseudotime
Pseudotime per celltype
Monocle3 Pseudotime
E
GTPase regulator activity
small GTPase−mediated signal transduction
regulation of protein−containing complex assembly
muscle cell differentiation
GTP binding
GTPase activity
vascular process in circulatory system
regulation of muscle system process
regulation of cell morphogenesis
interferon−mediated signaling pathway
mesenchymal cell migration
regulation of mitophagy
G protein activity
GeneRatio
p.adjust
F
MAPK signaling pathway
Calcium signaling pathway
muscle system process
cGMP−PKG signaling pathway
regulation of metal ion transport
Ras signaling pathway
Rap1 signaling pathway
muscle cell differentiation
actin filament organization
Cytoskeleton in muscle cells
Phospholipase D signaling pathway
regulation of protein kinase activity
cAMP signaling pathway
response to oxygen levels
Gap junction
mesenchyme development
Vascular smooth muscle contraction
Notch signaling pathway
negative regulation of cell adhesion
G
Expression level
Prkg1
Cacna1c
Itgb1
Rap1b
Nfkb1
H
cGMP
Ca2+ transport
Integrin
Rap1
MAPK
Hedgehog
Score
ρ: 0.8192986 p-value < 2.2E-16
ρ: 0.7367516 p-value < 2.2E-16
ρ: 0.6775952 p-value < 2.2E-16
ρ: 0.6571958 p-value < 2.2E-16
ρ: 0.4867923 p-value < 2.2E-16
ρ: 0.290013 p-value = 1.445E-14
Monocle3 Pseudotime
I
tdT+ Myh11+
tdT- Myh11+
Percent Expressed
Average Expression
Cd59a
Plxna4
Nalcn
Npnt
Ntrk3
Fblim1
tdTomato
Steap4
Agmo
Adam12
Rgs16
Cxcl1
J
regulation of hydrolase activity
muscle cell differentiation
wound healing
contractile muscle fiber
actin filament organization
actin binding
regulation of actin filament−based process
collagen−containing extracellular matrix
cell−substrate adhesion
regulation of smooth muscle cell proliferation
regulation of muscle contraction
regulation of angiogenesis
potassium ion transport
morphogenesis of a branching epithelium
coagulation
positive regulation of transforming growth factor beta receptor signaling pathway
positive regulation of MAP kinase activity
focal adhesion
GeneRatio
p.adjust
K
Acta2
Tagln
Cnn1
Myh11
Vcl
Des
Myl9
Cald1
Mylk
Cacna1c
Ryr2
Itpr1
Expression Level
tdT+ Myh11+
tdT- Myh11+

Figure S4. ScRNA-seq analysis of the inner mechanisms of Gli1^{+} cells trans-differentiating into SMCs

(A) UMAP visualization of unsupervised clustering for tdTomato^{+} (tdT^{+}) fibroblasts and SMCs isolated from the injured femoral artery (FA) of tamoxifen-treated *Gli1-CreER;R26-tdT* mice.

(B, C) Pseudotime trajectory analysis was used to infer the trans-differentiation of tdT^{+} Gli1^{+} ASCs into SMCs.

(D-F) The GO and KEGG analysis of the enriched gene modules(D) for the SMC1 cluster (E) and SMC2 cluster (F).

(G) Violin plots depicting the expression levels of key signaling genes across clusters: cGMP signaling (*Prkg1*), Ca^{2+} transport (*Cacna1c*), Integrin signaling (*Itgb1*), Rap1 signaling (*Rap1b*), and MAPK signaling (*Nfkb1*).

(H) Spearman's rank correlation analysis of signaling pathway activity scores along the pseudotime trajectory.

(I, J) Analysis of Myh11^{+} tdT^{-} and Myh11^{+} tdT^{+} cells from injured FA, including (I) differentially expressed genes (DEGs) and (J) associated pathway enrichment.

(K) Violin plots showing the expression of structural and functional SMC markers: contractile markers (*Acta2*, *Tagln*, *Cnn1*, *Myh11*), cytoskeletal organization (*Vcl*, *Des*), contractile regulation (*Myl9*), myosin phosphorylation (*Cald1*, *Mylk*), and Ca^{2+} signaling (*Cacna1c*, *Ryr2*, *Itpr1*).

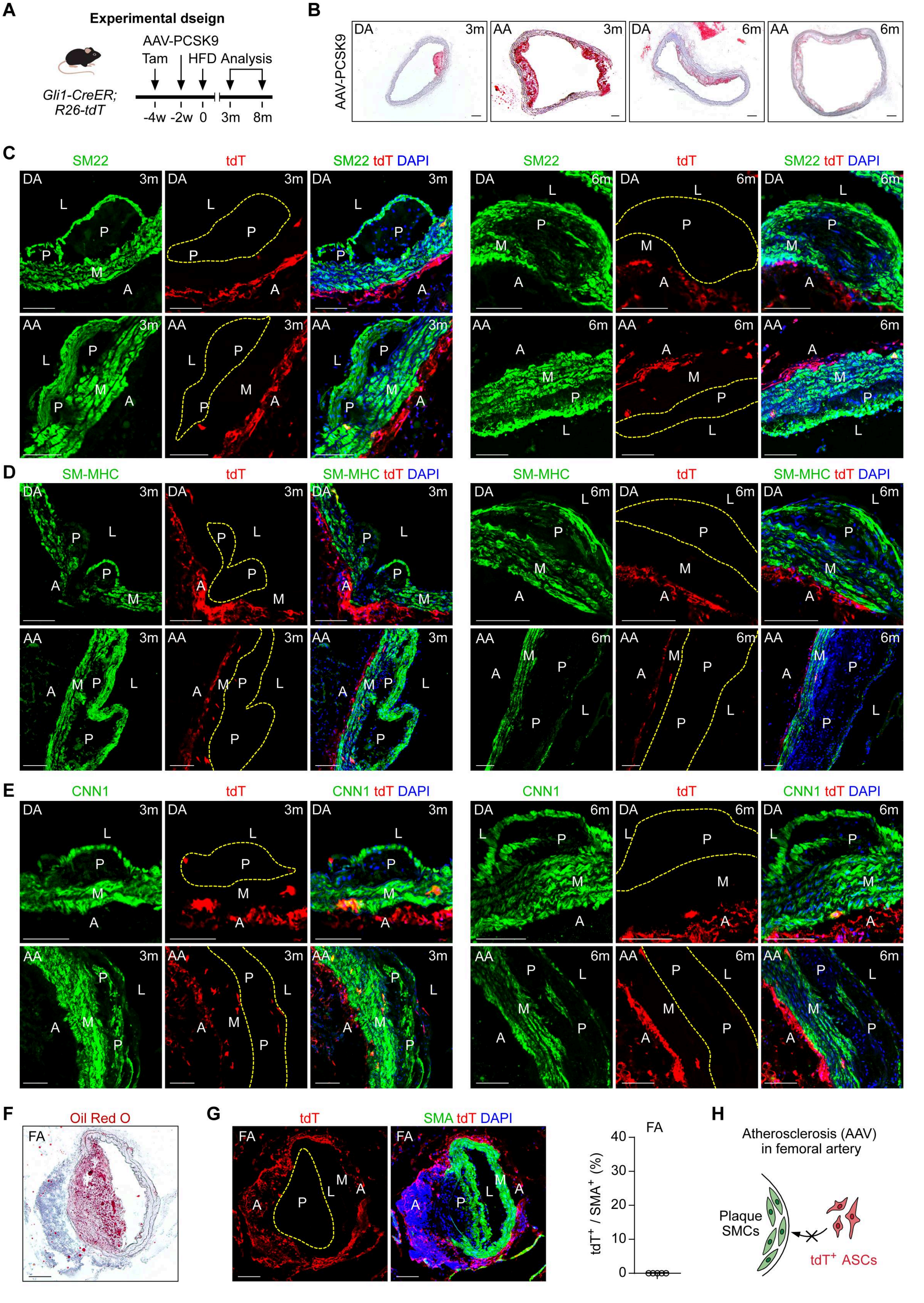
A
Experimental dseign
Gli1-CreER; R26-tdT
AAV-PCSK9
Tam
HFD Analysis
-4w -2w 0 3m 8m
B
AAV-PCSK9
DA 3m
AA 3m
DA 6m
AA 6m
C
SM22
tdT
SM22 tdT DAPI
D
SM-MHC
tdT
SM-MHC tdT DAPI
E
CNN1
tdT
CNN1 tdT DAPI
F
Oil Red O
FA
G
tdT
SMA tdT DAPI
FA
tdT+ / SMA+ (%)
0
10
20
30
40
H
Atherosclerosis (AAV) in femoral artery
Plaque SMCs
tdT+ ASCs

Figure S5. tdT$^+$ ASCs minimally transform into SMCs in AAV-induced atherosclerosis.
(A) Schematic diagram showing the experimental design. Tam: Tamoxifen, HFD: high-fat diet.
(B) Oil red O staining results of DA and AA from AAV-PCSK9 injection-treated *Gli1-CreER;R26-tdT* mice with 3 months (3m) and 6 months (6m) HFD.
(C-E) Immunostaining results of atherosclerotic DA and AA sections with mutiple SMCs antibodies such as SM22 (C), SM-MHC (D) and CNN1 (E). A: adventitia, M: media, P: plaque, L: lumen. The yellow dotted circle marks the area of atherosclerosis plaque.
(F) Oil red O staining results of FA sections from AAV-PCSK9 injection-treated *Gli1-CreER;R26-tdT* mice with 8m HFD.
(G) Immunostaining and statistics results of atherosclerotic FA sections with SMA antibody.
(H) Cartoon figure summarized the tdT$^+$ ASCs did not transform into SMCs in plaque of AAV-induced femoral artery atherosclrosis, n=5.
White and black scale bars: 100μm.

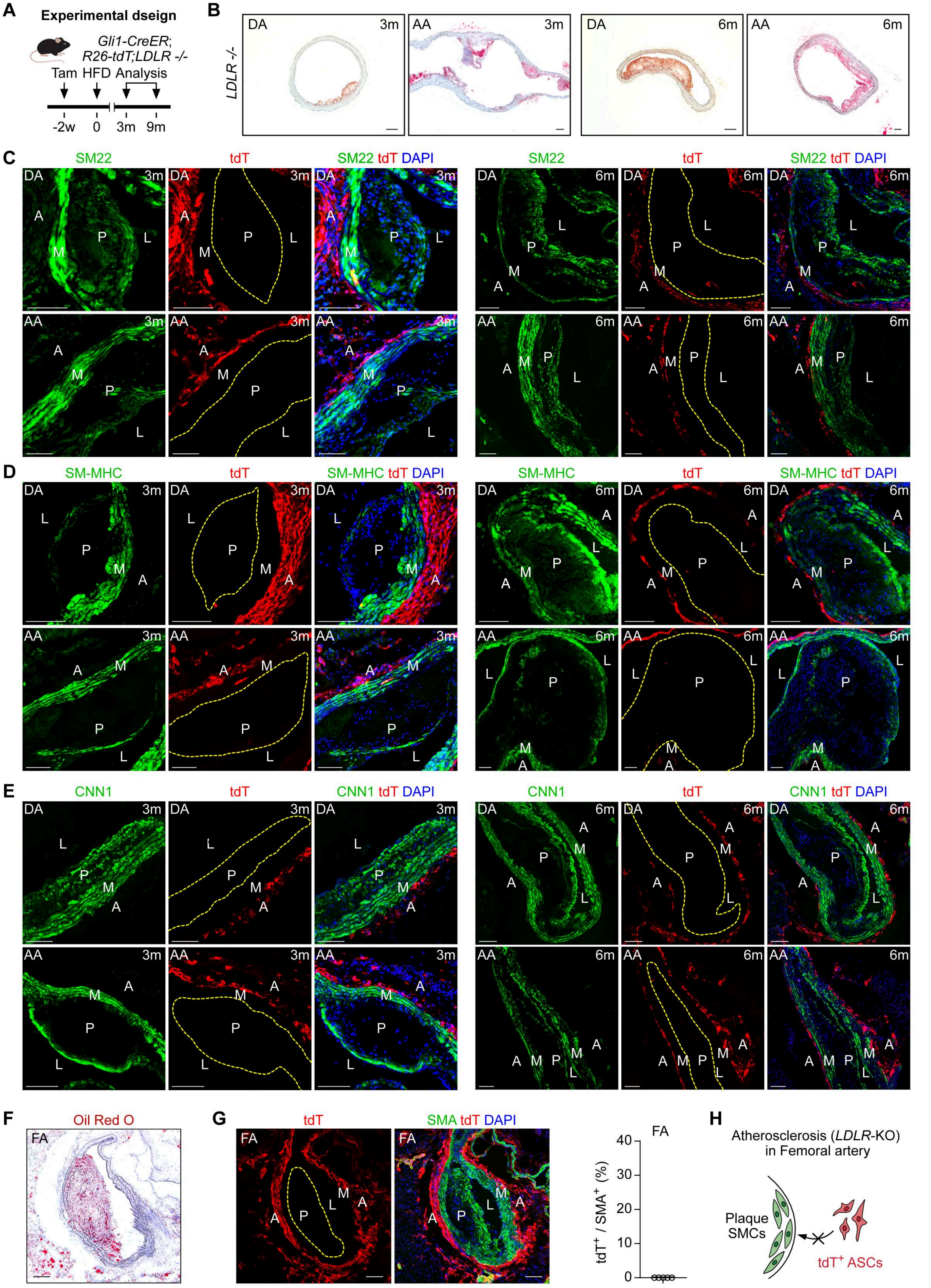
A
Experimental dseign
Gli1-CreER; R26-tdT;LDLR -/-
Tam HFD Analysis
-2w 0 3m 9m
B
LDLR -/-
DA 3m
AA 3m
DA 6m
AA 6m
C
SM22
tdT
SM22 tdT DAPI
D
SM-MHC
tdT
SM-MHC tdT DAPI
E
CNN1
tdT
CNN1 tdT DAPI
F
Oil Red O
FA
G
tdT
SMA tdT DAPI
FA
tdT+ / SMA+ (%)
0
10
20
30
40
H
Atherosclerosis (LDLR-KO) in Femoral artery
Plaque SMCs
tdT+ ASCs

Figure S6: tdT$^+$ ASCs hardly transform into SMCs in *LDLR* KO-induced atherosclerosis.
(A) Schematic diagram showing the experimental design. Tam: Tamoxifen, HFD: high-fat diet.
(B) Oil red O staining results of DA and AA from *Gli1-CreER;R26-tdT;LDLR-/-* mice with 3 months (3m) and 6 months (6m) HFD.
(C-E) Immunostaining results of atherosclerotic DA and AA sections with mutiple SMCs antibodies such as SM22 (C), SM-MHC (D) and CNN1 (E).
A: adventitia, M: media, P: plaque, L: lumen. The yellow dotted circle marks the area of atherosclerosis plaque.
(F) Oil red O staining results of FA sections from *Gli1-CreER;R26-tdT;LDLR-/-* mice with 9m HFD.
(G) Immunostaining results of atherosclerotic FA sections with SMA antibody.
(H) Cartoon figure summarized the tdT$^+$ ASCs did not transform into SMCs in plaque of *LDLR* KO-induced atherosclrosis.
White and black scale bars: 100 μm.

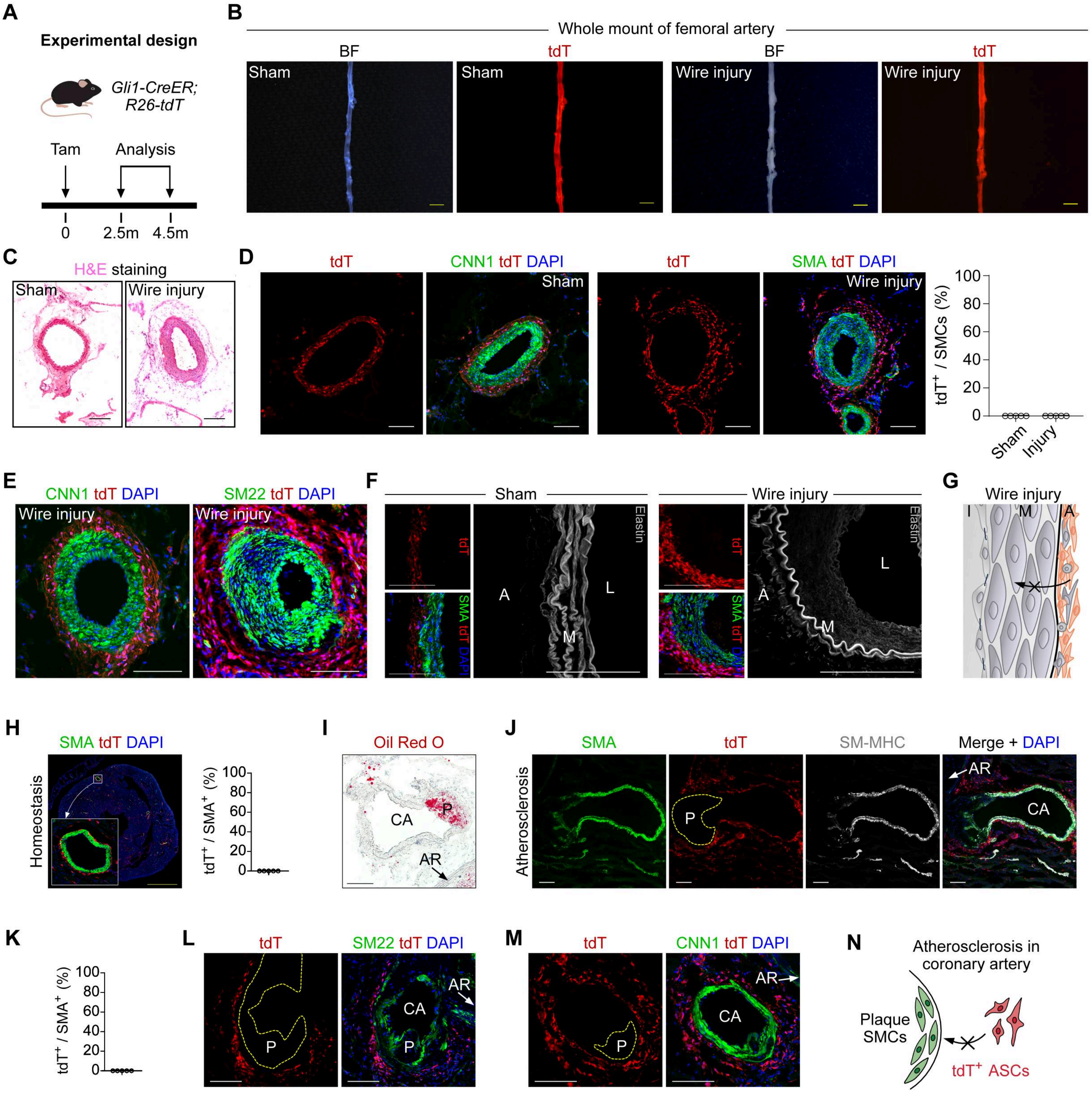

Figure S7. tdT^+ ASCs do not contribute to SMCs in femoral artery wire injury and coronary artery atherosclerosis.
(A) Schematic diagram showing the experimental design. Tam: Tamoxifen.
(B) Whole-mount of sham and wire injury FA from *Gli1-CreER;R26-tdT* mice with Tam treatment.
(C) H&E staining results of sham and wire injury FA sections.
(D) Immunostaining results of sham and wire injury FA sections with SMCs and tdT antibodies, n=5.
(E) Immunostaining results of wire injury FA sections with SMCs and tdT antibodies.
(F) Immunostaining results of sham and wire injury FA sections with SMA, Elastin and tdT antibodies.
(G) Cartoon figure showing tdT^+ ASCs do not transform into SMCs in FA under wire injury conditions.
(H) Immunostaining and statistics results of heart sections from Tam-treated *Gli1-CreER;R26-tdT* mice SMA and tdT antibodies, n=5.
(I-K) Oil Red O staining and immunostaining results of coronary artery (CA) sections from Tam-treated and AAV-PCSK9 injected *Gli1-CreER;R26-tdT* mice with 8-9 months HFD. Statistics results come from immunostaining pictures, n=5.
(L, M) Immunostaining results of coronary artery (CA) sections from Tam-treated and AAV-PCSK9 injected *Gli1-CreER;R26-tdT* mice with 8-9 months HFD using SM22, CNN1 and tdT antibodies.
(N) Cartoon figure showing tdT^+ ASCs do not transform into SMCs in CA under atherosclerosis conditions.
Yellow scale bars: 1000 μm. Black and white scale bars: 100 μm.

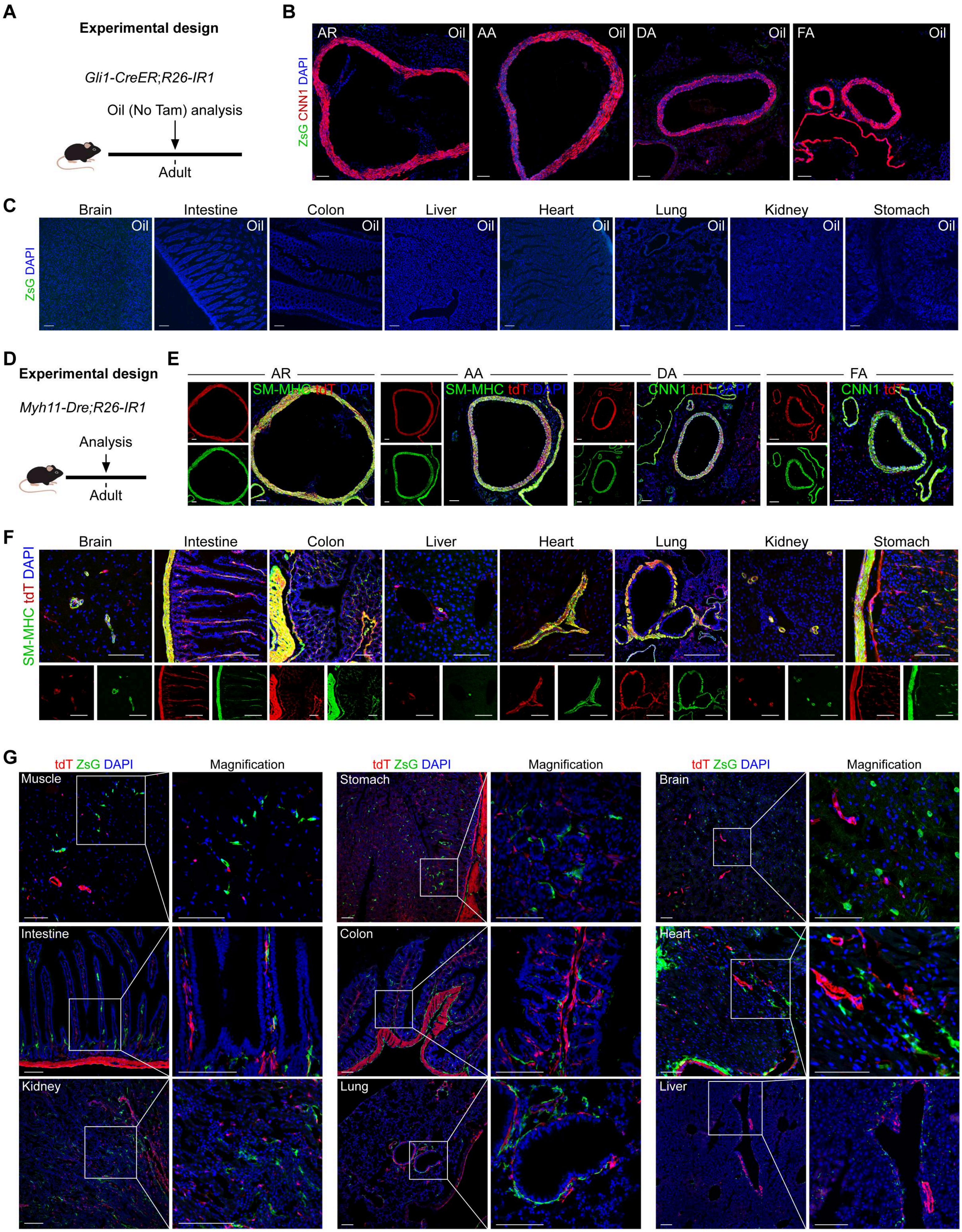
A
Experimental design
Gli1-CreER;R26-IR1
Oil (No Tam) analysis
Adult
B
ZsG CNN1 DAPI
AR
AA
DA
FA
Oil
C
Brain
Intestine
Colon
Liver
Heart
Lung
Kidney
Stomach
ZsG DAPI
D
Experimental design
Myh11-Dre;R26-IR1
Analysis
Adult
E
AR
AA
DA
FA
SM-MHC tdT DAPI
CNN1 tdT DAPI
F
SM-MHC tdT DAPI
G
tdT ZsG DAPI
Magnification
Muscle
Intestine
Kidney
Stomach
Colon
Lung
Brain
Heart
Liver

Figure S8. Characterization of *Myh11-Dre;Gli1-CreER;R26-IR1* mice.
(A) Schematic diagram of experimental design for 30w-old *Gli1-CreER;R26-IR1* mice.
(B, C) Immunostaining results of arteries (B) and other organs (C) of *Gli1-CreER;R26-IR1* mice.
(D) Schematic diagram of experimental design for 30w-old *Myh11-Dre;R26-IR1* mice
(E, F) Immunostaining results of aortas (E) and other organs (F) of *Myh11-Dre;R26-IR1* mice.
(G) Immunostaining results of multiple organs of *Myh11-Dre;Gli1-CreER; R26-IR1* mice with Tam treatment demonstrating no co-location of tdT and ZsG.
White scale bars: 100 μm.

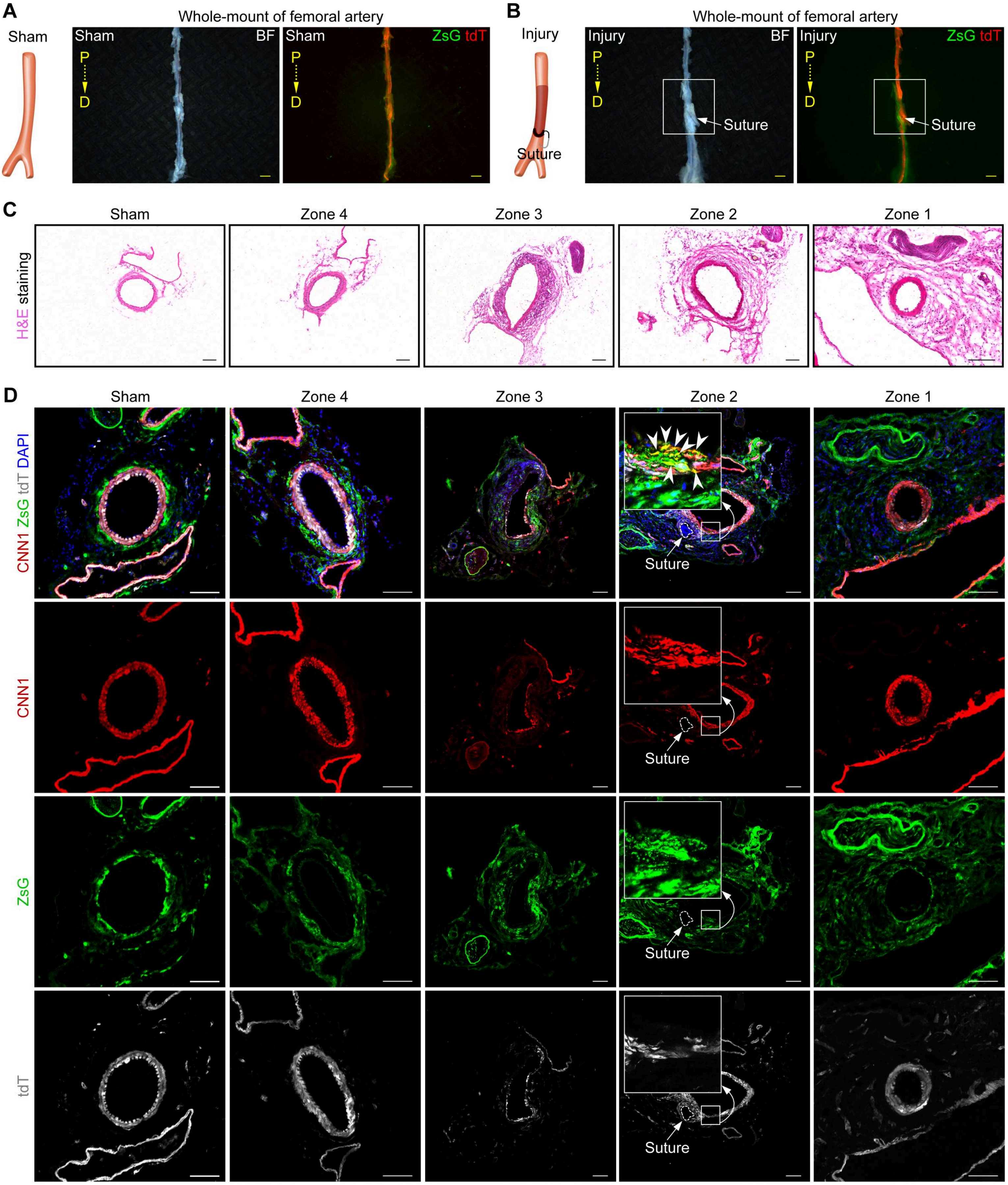

Figure S9. ZsG$^+$ ASCs contribute to SMCs in anastomosis injury of femoral artery.
(A-B) Whole-mount results of the injury and the sham FA. BF: bright-field. P: proximal, D: distal. Cartoon figure illustrating the anastomosis injury of FA and the sham.
(C) H&E staining results of the sham FA sections and Zone 1-Zone 4 of the injury FA sections.
(D) Immunostaining results of the sham FA sections and Zone 1-Zone 4 of the injury FA sections with CNN1 antibody.
White and black scale bars: 100 μm, yellow scale bars: 1000 μm.

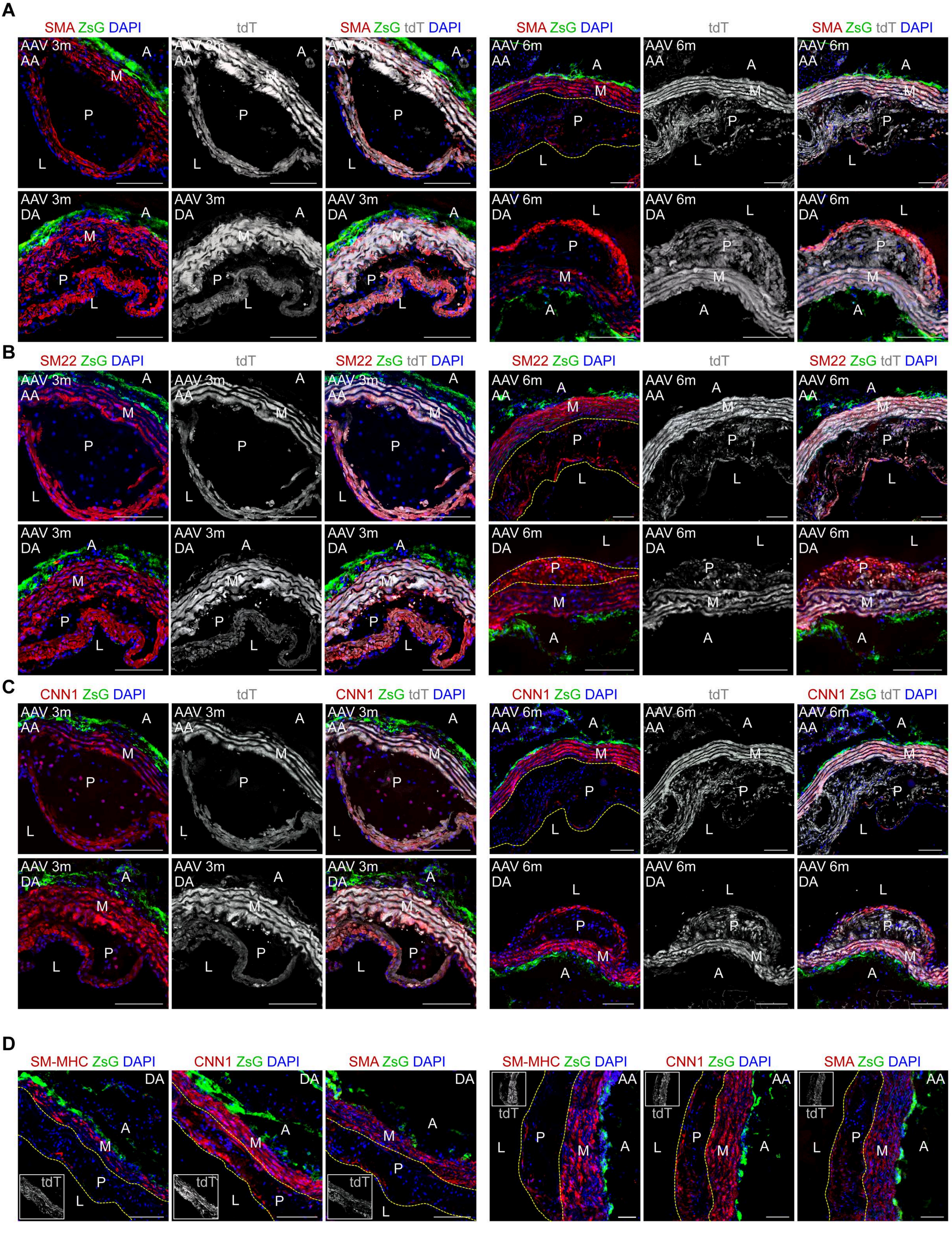

Figure S10. ZsG+ ASCs do not transform into SMCs in plaque of AA and DA.

(A-C) Immunostaining results of 3m- and 6m-HFD AA and DA sections from *Myh11-Dre;Gli1-CreER;R26-IR1* mice with AAV-PCSK9 (AAV) injection.

(D) Immunostaining results of 14w-HFD AA and DA sections from *Myh11-Dre;Gli1-CreER;R26-IR1;LDLR -/-* mice.

White scale bars: 100 μm.

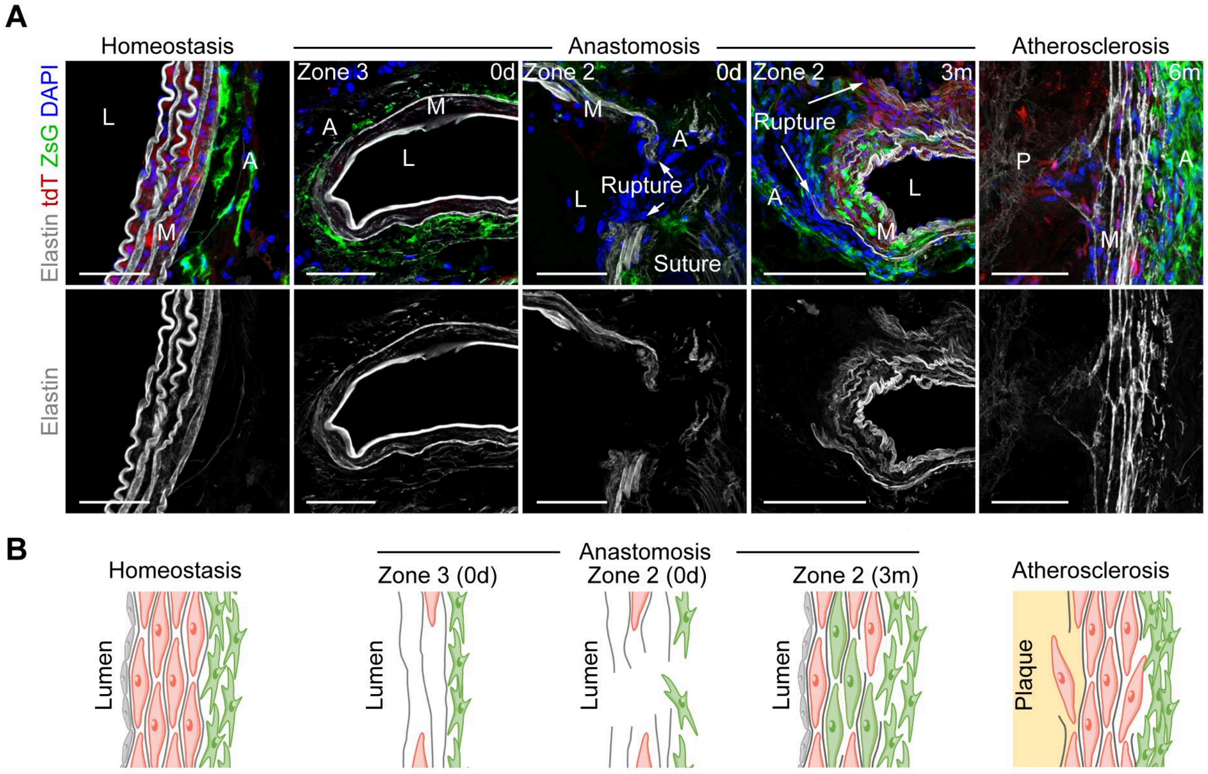

Figure S11. Distinct lineage contributions of ZsG$^+$ ASCs in the vasculature during homeostasis, anastomosis injury, and atherosclerosis.
(A) Representative immunofluorescence images of arterial sections from tamoxifen-induced *Myh11-Dre;Gli1-CreER;R26-IR1* mice under homeostasis, following anastomosis injury, or in an atherosclerosis model, stained for Elastin and ZsG.
(B) Schematic diagrams summarizing the experimental paradigms corresponding to each condition presented in (A).
White scale bars: 100 μm.
